# Chronic Stress‐Induced and Tumor Derived SP1^+^ Exosomes Polarizing IL‐1β^+^ Neutrophils to Increase Lung Metastasis of Breast Cancer

**DOI:** 10.1002/advs.202310266

**Published:** 2024-12-04

**Authors:** Leyi Zhang, Jun Pan, Meijun Wang, Jini Yang, Sangsang Zhu, Lili Li, Xiaoxiao Hu, Zhen Wang, Liwei Pang, Peng Li, Fang Jia, Guohong Ren, Yi Zhang, Danying Xu, Fuming Qiu, Jian Huang

**Affiliations:** ^1^ Department of Breast Surgery Second Affiliated Hospital Zhejiang University School of Medicine 88 Jiefang Road Hangzhou Zhejiang 310009 China; ^2^ Key Laboratory of Tumor Microenvironment and Immune Therapy of Zhejiang Province Second Affiliated Hospital Zhejiang University School of Medicine Hangzhou Zhejiang 310009 China; ^3^ Cancer Center Zhejiang University Hangzhou Zhejiang 310009 China; ^4^ Cancer Institute (Key Laboratory of Cancer Prevention & Intervention National Ministry of Education) Second Affiliated Hospital Zhejiang University School of Medicine Hangzhou Zhejiang 310009 China; ^5^ Department of Medical Oncology Second Affiliated Hospital Zhejiang University School of Medicine Hangzhou Zhejiang 310009 China; ^6^ Department of Breast surgery The Second Hospital of Jiaxing Jiaxing Zhejiang 314000 China

**Keywords:** breast cancer, chronic stress, exosomes, lung metastasis, neutrophils

## Abstract

Chronic stress can significantly promote breast cancer progression. When exposed to chronic stress, exosomes released from neural and neuroendocrine cells in the central nervous system are enhanced and modified. However, whether tumor‐derived exosomes (TDEs) are influenced by chronic stress and participate in chronic stress‐mediated distant metastasis remains unclear. Here, it is shown that chronic stress remarkably facilitates the secretion of TDEs and modifies the contents of exosomes by activating the adrenergic β receptor in 4T1 tumor‐bearing mice. Exosomes injection and blockade experiments indicate that exosomes play a crucial role in chronic stress‐mediated lung metastasis of breast cancer. Chronic stress‐induced TDEs are internalized by pulmonary neutrophils and strengthen neutrophil recruitment via the CXCL2 autocrine. In addition, the level of SP1 in TDEs increases, which favors the secretion of IL‐1β by neutrophils through the activation of the TLR4‐NFκβ pathway, ultimately aggravating lung metastasis of breast cancer. Collectively, this study provides a novel mechanism by which neutrophils within a pre‐metastatic niche acquire their inflamed phenotype and establishes an important link among neuroendocrine changes, exosomes, immunity, and metastasis.

## Graphical abstract

Chronic stress significantly enhances the secretion of SP1^+^ tumor‐derived exosomes through the adrenergic β receptor. This not only strengthens neutrophil self‐recruitment via the CXCL2 autocrine but also promotes neutrophil secretion of IL‐1β through the TLR4‐NFκβ pathway, thereby remodeling the lung microenvironment and contributing to the promotion of lung metastasis of breast cancer.

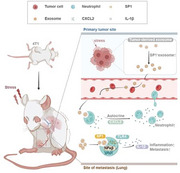

## 1 Introduction

Neuroendocrine dysfunction induced by chronic stress is one of the important factors promoting the occurrence and metastasis of cancer, including breast cancer. Chronic stress significantly promotes tumor progression by enhancing the stemness, colonization, and angiogenesis of tumor cells.^[^ ^1^ ^]^

Metastatic breast cancer has a high mortality rate, with a 5‐year survival rate of only 27%.^[^ ^2^ ^]^ Among them, triple‐negative breast cancer is highly invasive and prone to metastasizing to organs such as the lungs.^[^ ^3^ ^]^ Breast cancer can reprogram the lung microenvironment to generate pre‐metastatic niches and support the colonization of disseminated tumor cells.^[^ ^4^ ^]^ Breast tumor‐derived exosomes (TDEs) can be transported to the lungs through the bloodstream, fusing with target cells in the lungs to deliver their cargos and remodeling an immune‐suppressive microenvironment.^[^ ^5^ ^]^

Neutrophils are known to promote breast cancer lung metastasis by forming neutrophil extracellular traps (NETs) that capture disseminated tumor cells and suppress cytotoxic T lymphocytes; thus, accelerating the establishment of metastases.^[^ ^6^ ^]^ Nevertheless, its contribution to chronic stress‐mediated metastasis remains unclear. Long‐term stress has been reported to activate upstream hematopoietic stem cells, leading to an increased output of neutrophils,^[^ ^7^ ^]^ which suggests chronic stress may promote breast cancer metastasis via neutrophils.

Exosomes‐mediated transfer of biologically active substances from source cells to neutrophils remodels the chemotaxis of neutrophils as well as their functions in NET formation and release of inflammatory factors.^[^ ^8^ ^]^

Chronic stress modifies the content and enhances the release of exosomes from neural and neuroendocrine cells in the central nervous system.^[^ ^9^ ^]^ However, the role of tumor‐derived extracellular vesicles in chronic stress‐mediated breast cancer lung metastasis remains unclear. In our study, we found that chronic stress‐induced neuroendocrine changes affected TDEs, promoting neutrophils to secrete C‐X‐C Motif Chemokine Ligand 2 (CXCL2), which enhanced recruitment. After chronic stress, the level of Sp1 Transcription Factor (SP1) in TDEs increased, facilitating neutrophil secretion of Interleukin 1 Beta (IL‐1β) through the Toll‐Like Receptor 4 (TLR4)‐Nuclear Factor Kappa B (NFκβ) pathway, which created an immune‐suppressive microenvironment and aggravated breast cancer lung metastasis.

## 2 Results

### 2.1 Chronic Stress Enhances and Modifies Breast TDEs in 4T1 Tumor‐Bearing Mice

**Figure 1:**
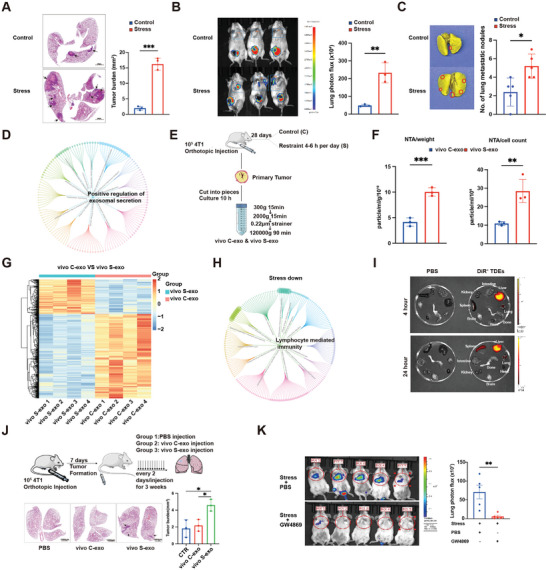
Chronic stress alters breast tumor tissue‐derived exosomes and promotes lung metastasis. A) Representative H&E staining and quantification of lung metastases in 4‐week tumor‐bearing mice from the control group (*n* = 3) and the chronic stress group (*n* = 3). Scale bar: 1 mm. B) Bioluminescent image and quantitative analysis of the lungs of mice injected with luciferase‐expressing 4T1 tumor cells orthotopically in the control group (*n* = 3) and the stress group (*n* = 3), 4 weeks post‐injection. C) Representative Bouin's staining and quantification of lung metastatic nodules in 4‐week tumor‐bearing mice from the control group (*n* = 5) and the chronic stress group (*n* = 5). D) Gene ontology analysis of up‐regulated genes in the chronic stress group from the GSE154685 dataset. Terms with a *p* value < 0.05 are shown. E) Diagram illustrating the isolation of exosomes from the culture supernatant of tumor tissues from 4‐week tumor‐bearing mice. F) Concentrations of vivo C‐exo (*n* = 3) and vivo S‐exo (*n* = 3) were measured by NTA, calibrated by the weight of tumor tissues and the cell counts of digested tumor tissues. G) Heatmap displaying differentially expressed proteins (*p* < 0.05, fold change ≥ 1.2 or ≤0.8) from the proteome analysis of vivo C‐exo and vivo S‐exo. H) Gene ontology analysis of down‐regulated proteins in the proteome analysis of vivo S‐exo. I) Representative images of the indicated organs for detection of DiR‐labeled TDEs at 4 and 24 h after injection. J) Diagram illustrating the exosome injection experiment via the mouse tail vein. 200 µL PBS, 20 µg of vivo C‐exo in 200 µL PBS, and 20 µg of vivo S‐exo in 200 µL PBS were respectively injected into BALB/C mice that had been subcutaneously injected with 10^5^ 4T1 tumor cells 7 days prior (upper). Representative H&E staining and quantification of lung metastases in 4‐week tumor‐bearing mice from the control group (*n* = 3), the vivo C‐exo injection group (*n* = 3), and the vivo S‐exo injection group (*n* = 3) (lower). K) Representative bioluminescent image and quantitative analysis of the lungs of mice injected with luciferase‐expressing 4T1 tumor cells via the tail vein in the stress + PBS injection group (*n* = 5) and the stress + GW4869 injection group (*n* = 5), 2 weeks post‐injection. The data are shown as mean ± SEM. *: *p* < 0.05, **: *p* < 0.01, and ***: *p* < 0.001.

Chronic stress has been known to significantly promote breast cancer lung metastasis. We exposed 4T1 tumor‐bearing wildtype BALB/C mice to chronic restraint stress for 28 consecutive days and designated them as the stress group.^[^ ^10^ ^]^ The 4T1 tumor‐bearing mice with no treatment were designated as the control group. The lung metastasis of breast cancer was accelerated by chronic stress (**Figure** **1**A–C; Figure S1A,B, Supporting Information). We re‐analyzed the GSE154685 dataset, which had a similar model to ours, and found that chronic restraint stress notably altered the expression levels of genes related to exosomes (Figure S1C, Supporting Information).^[^ ^1c^ ^]^ We performed a gene ontology (GO) analysis of the up‐regulated genes in the stress group, which suggested the notion that chronic stress positively regulated exosomal secretion of tumor (Figure 1D). To verify this notion, we isolated TDEs and evaluated their purity (Figure 1E; Figure S1D–G, Supporting Information). Quantificationally, we found that chronic stress significantly promoted TDE secretion (Figure 1F; Figure S1H, Supporting Information). To further explore whether chronic stress influenced the contents of TDEs, we performed proteomic analysis on TDEs from the control group (vivo C‐exo) and TDEs from the stress group (vivo S‐exo). Principal component analysis and heatmap representation clearly showed a considerable difference in the expression patterns and contents of vivo C‐exo and vivo S‐exo (Figure 1G; Figure S1I, Supporting Information). The GO analysis of down‐regulated proteins in vivo S‐exo revealed immune suppression (Figure 1H; Figure S1J, Supporting Information). An analysis of protein–protein interactions (PPI) for the up‐regulated proteins in vivo S‐exo was carried out (Figure S2, Supporting Information). Collectively, these data suggested that chronic stress modified TDEs quantitatively and qualitatively.

We next aimed to determine whether chronic stress‐induced TDEs affected the progression of breast cancer. In vivo and ex vivo fluorescent images showed that liver, lung, and spleen were the dominant targets of DiR‐labeled TDEs with high fluorescence intensity (Figure 1I; Figure S1K, Supporting Information). 4T1 tumor‐bearing mice treated with vivo S‐exo had an increased number of lung metastases, as assessed by hematoxylin and eosin (H&E) staining (Figure 1J). To further assess the effects of TDEs, we used GW4869, a widely used potent neutral sphingomyelinase inhibitor that was able to block exosome production.^[^ ^11^ ^]^ Blocking exosome production under chronic stress significantly reduced lung metastasis of breast cancer (Figure 1K; Figure S1L, Supporting Information). Altogether, these results suggested that chronic stress‐induced TDEs played a pivotal role in promoting lung metastasis of breast cancer.

### 2.2 Identification of the β‐adrenergic Receptor as a Sensor for Elevating Chronic Stress‐Induced Exosome Secretion

**Figure 2:**
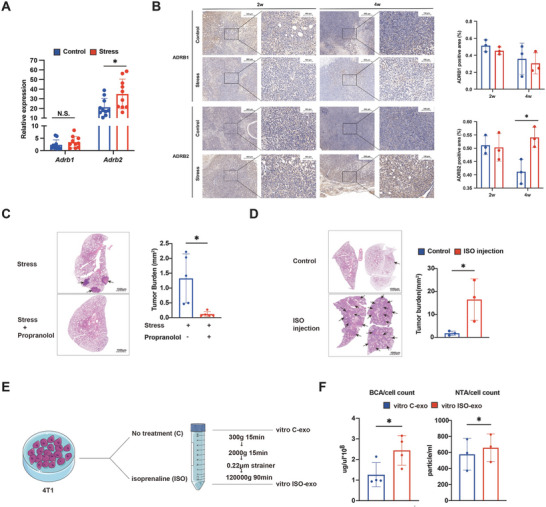
Elevated β‐adrenergic receptor expression increased exosome secretion and lung metastasis. A) qRT‐PCR analysis of *Adrb1* and *Adrb2* expression in total tumor mass from 4‐week tumor‐bearing mice in the control group (*n* = 10) and the chronic stress group (*n* = 10). B) Quantification of ADRB1 and ADRB2 immunohistochemical staining in tumor tissues from 2‐week and 4‐week tumor‐bearing mice in the control group (*n* = 3) and the chronic stress group (*n* = 3). C) Representative H&E staining and quantification of lung metastases in 4‐week tumor‐bearing mice from the chronic stress group (*n* = 5) and the chronic stress and propranolol co‐treated group (*n* = 5). Scale bar: 1 mm. D) Representative H&E staining and quantification of lung metastases in 4‐week tumor‐bearing mice from the PBS‐injected group (*n* = 3) and the ISO‐injected group (*n* = 3). Scale bar: 1 mm. E) Diagram showing exosomes isolated from the culture supernatant of 4T1 cells in the control group and ISO‐treated group. F) The concentrations of vitro C‐exo and vitro ISO‐exo were measured by BCA method (*n* = 4) and NTA (*n* = 3) and calibrated by cell counts. The data are shown as mean ± SEM. *: *p* < 0.05 and **: *p* < 0.01.

The activatio of sustained adrenergic signaling under chronic stress has been reported. Our results showed that the expression levels of adrenoceptor beta 2 (ADRB2), not of adrenoceptor beta 1 (ADRB1), were elevated in tumor tissues when exposed to chronic stress, as stated in previous study (**Figure** **2**A).^[^ ^12^ ^]^ Further, the expression levels of ADRB2 were significantly higher until 4 weeks after implantation (Figure 2B). Treatment with the β‐adrenergic receptor blocker propranolol effectively restrained chronic stress‐mediated lung metastasis of breast cancer (Figure 2C); while, injection of the β‐adrenergic receptor agonist isoproterenol (ISO) promoted lung metastasis in breast tumor‐bearing mice (Figure 2D). However, the use of propranolol did not inhibit the growth of primary tumors (Figure S3A, Supporting Information). These data verified the pivotal role of adrenergic signaling in chronic stress‐induced metastasis.

It has been reported that norepinephrine, one of the catecholamines, can boost the release of exosomes from mesenchymal stem cells and fibroblasts.^[^ ^13^ ^]^ We speculated that chronic stress boosted exosome secretion by stimulating the β‐adrenergic receptor of tumor cells. The 4T1 cells were co‐cultured with ISO to simulate chronic stress in vitro. Then, we collected and verified exosomes derived from the 4T1 cells co‐cultured with or without ISO treatment (Figure 2E; Figure S3B–D, Supporting Information). Using nanoparticle tracking analysis (NTA) and bicinchoninic acid (BCA) assays, we found that adrenergic receptor activation notably promoted exosome secretion of tumor cells (Figure 2F). Together, these data suggested that elevated β‐adrenergic receptor expression was implicated in chronic stress‐induced expedited exosome secretion and lung metastasis.

### 2.3 TDEs are Internalized by Pulmonary Neutrophils and Strengthen Their Recruitment Via CXCL2 Autocrine

**Figure 3:**
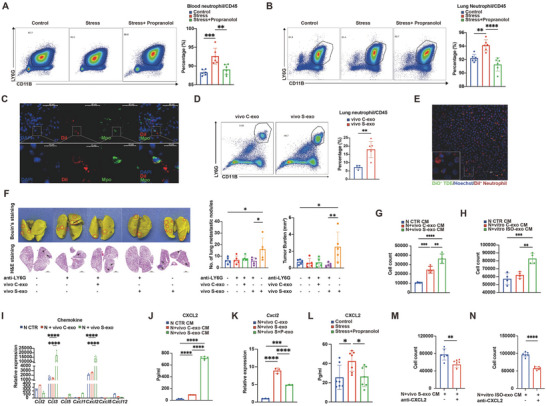
Chronic stress strengthens neutrophil self‐recruitment via CXCL2. Representative images of flow cytometry and quantification of A) PB‐infiltrating and B) lung‐infiltrating neutrophils in 4‐week tumor‐bearing mice from the control group (*n* = 6), chronic stress group (*n* = 6), and chronic stress and propranolol co‐treated group (*n* = 6). C) Representative lung immunofluorescence staining of mice injected with DiI^+^ vivo S‐exo. Red indicates DiI^+^ exosomes, blue indicates DAPI, and green indicates MPO. Scale bar: 10 µm (bottom) and 40 µm (up). D) Representative images of flow cytometry and quantification of lung‐infiltrating neutrophils 4 h after injection with DiI^+^ vivo C‐exo (*n* = 5) and vivo S‐exo (*n* = 5). E) Representative image of DiI‐labeled neutrophils absorbing DiO‐labeled TDEs after 4 h of co‐culture. 488 nm, TDE; 405 nm, Hoechst; 555 nm, neutrophils. The arrows indicate neutrophils that have taken up DiO‐labeled exosomes. F) Representative images of Bouin's and H&E staining for lungs from the 4‐week tumor‐bearing mice. Quantification of the number of lung metastatic nodules and burden of lung metastases (*n* = 5 mice/group). G,H) Transwell migration assay to detect the neutrophil recruitment ability of the conditioned medium (*n* = 4). I) qRT‐PCR analysis of chemokine expression in naïve neutrophils treated alone (*n* = 3), with vivo C‐exo (*n* = 3), and vivo S‐exo (*n* = 3), respectively for 4 h. J) ELISA analysis of CXCL2 concentrations in the supernatant of naïve neutrophils treated alone (*n* = 5), with vivo C‐exo (*n* = 5), and vivo S‐exo (*n* = 5), respectively for 4 h. K) qRT‐PCR analysis of *Cxcl2* expression in naïve neutrophils treated with vivo C‐exo (*n* = 3), vivo S‐exo (*n* = 3), and vivo S+P‐exo (*n* = 3) for 4 h. L) ELISA analysis of CXCL2 concentrations in the lung supernatant of 4‐week tumor‐bearing mice in the control group (*n* = 7), chronic stress group (*n* = 7), and chronic stress and propranolol co‐treated group (*n* = 7). M,N) Transwell migration assay to detect the neutrophil recruitment ability of N+vivo S‐exo CM (*n* = 6) and N+vitro ISO‐exo CM (*n* = 6) with or without the addition of anti‐CXCL2 antibody. The data are shown as mean ± SEM. *: *p* < 0.05, **: *p* < 0.01, ***: *p* < 0.001, and ****: *p* < 0.0001.

Cancer‐immune dialogue has been discussed in the context of chronic stress.^[^ ^14^ ^]^ Stress‐mediated tumor progression has been partially attributed to immune dysregulation.^[^ ^15^ ^]^ Proteome analysis of vivo S‐exo showed immune suppression (Figure 1F). To investigate whether vivo S‐exo had immune‐modulatory effects, we first analyzed the peripheral blood (PB) and lung immune infiltrations in control mice, chronically stressed mice, and mice treated with chronic stress and propranolol (Figure S4A, Supporting Information). The numbers of circulating and pulmonary neutrophils increased in chronically stressed mice; while, propranolol treatment suppressed this transition (**Figure** **3**A,B). In order to explore the cause of neutrophilia, we injected DiI‐labeled vivo C‐exo and vivo S‐exo into BALB/C mice. Four hours later, DiI‐labeled exosomes could be detected in PB and lungs (Figure S4B, Supporting Information). Pulmonary immune cells accounted for most exosome absorption (Figure S4C, Supporting Information). Among them, neutrophils assimilated the highest number of exosomes. (Figure S4D, Supporting Information). To further examine the internalization of exosomes in neutrophils, fluorescent staining was performed, which showed the presence of DiI‐labeled exosomes within pulmonary neutrophils (Figure 3C). In addition, we noticed that the circulating and lung‐infiltrating neutrophils were significantly elevated after vivo S‐exo injection compared with vivo C‐exo injection (Figure 3D; Figure S4E, Supporting Information). We also cultured isolated DiI‐labeled pulmonary neutrophils together with DiO‐labeled TDEs in vitro, which further demonstrated that neutrophils could ingest TDEs (Figure 3E). We hypothesized that neutrophils were the effector cells of TDEs under chronic stress. We depleted neutrophils in tumor‐bearing mice injected with TDEs (Figure S4F, Supporting Information). In the neutrophil‐depleted mice, vivo S‐exo injection no longer increased lung metastasis, further demonstrating that neutrophils played a critical role in orchestrating chronic stress‐induced lung metastasis (Figure 3F). Together, these data indicated that neutrophils played a crucial role in orchestrating stress‐induced metastasis by responding to TDEs under chronic stress.

Next, we aimed to explore how TDEs under chronic stress may directly affect neutrophils and explain the increased recruitment of neutrophils. We collected supernatants from bone marrow‐derived neutrophils (BM‐PMNs) treated with either no additional reagents or exosomes in different states. It was shown that the supernatants from BM‐PMNs, co‐cultured with TDEs under chronic stress, increased the chemoattraction of BM‐PMNs (Figure 3G,H).

Aiming to identify the essential chemokines involved in the neutrophil recruitment caused by TDEs, we performed real‐time quantitative reverse transcription PCR (qRT‐PCR) analysis of BM‐PMNs treated with either no extra reagents or exosomes in different groups. The expression levels of *Ccl3* and *Cxcl2* were conspicuously up‐regulated in BM‐PMNs treated with vivo S‐exo (Figure 3I), which were also confirmed by ELISA (Figure 3J; Figure S4G, Supporting Information). However, propranolol significantly suppressed the promotive effects of stress‐induced TDEs on CXCL2 production; while, no difference in CCL3 production was observed (Figure 3K; Figure S4H, Supporting Information). We also measured the levels of chemokines in the lung culture supernatants from control mice, chronically stressed mice, and mice treated with chronic stress plus propranolol to examine this finding in vivo. As expected, the lung supernatant of chronically stressed mice had a higher level of CXCL2 than that of control mice, and propranolol weakened this trend (Figure 3L). Propranolol had no considerable effect on CCL3 levels (Figure S4I, Supporting Information). We also confirmed the promotive effects of vitro ISO‐exo on CXCL2 secretion by neutrophils (Figure S4J, Supporting Information). Then, we administered a CXCL2 neutralizing antibody, which attenuated the neutrophil chemotactic activity of the co‐culture supernatants, further proving the significance of CXCL2. The stimulative impacts of vivo S‐exo and vitro ISO‐exo were notably inhibited (Figure 3M,N). These data suggested that the internalized TDEs induced by chronic stress could strengthen neutrophil recruitment via CXCL2.

### 2.4 TDEs Polarize IL‐1β^+^ Neutrophils Through Activation of the TLR4‐NFκβ Pathway

**Figure 4:**
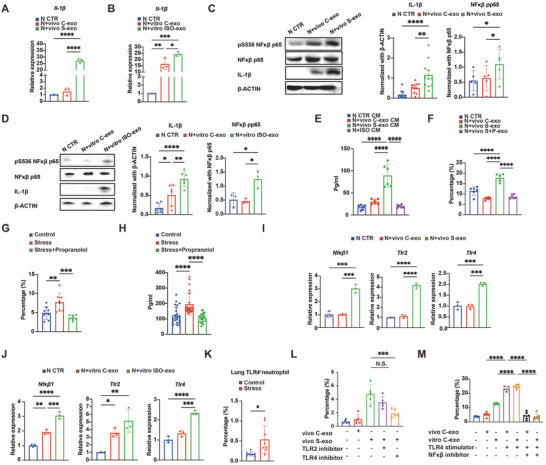
TDEs under chronic stress polarize IL‐1β^+^ neutrophils through the TLR4‐NFκβ pathway. A) qRT‐PCR analysis of *Il‐1β* expression in naïve neutrophils treated alone (*n* = 3), with vivo C‐exo (*n* = 3), and vivo S‐exo (*n* = 3), respectively for 4 h. B) qRT‐PCR analysis of *Il‐1β* expression of naïve neutrophils treated alone (*n* = 3), with vitro C‐exo (*n* = 3), and vitro ISO‐exo (*n* = 3), respectively for 4 h. C) WB analysis of IL‐1β (*n* = 11) and NFκβ pp65 (*n* = 5) expression in naïve neutrophils treated alone, with vivo C‐exo, and vivo S‐exo, respectively for 4 h. D) WB analysis of IL‐1β (*n* = 6) and NFκβ pp65 (*n* = 3) expression in naïve neutrophils treated alone, with vitro C‐exo, and vitro ISO‐exo, respectively for 4 h. E) ELISA analysis of IL‐1β concentrations in the supernatant of naïve neutrophils treated alone (*n* = 6), with vivo C‐exo (*n* = 6), vivo S‐exo (*n* = 6), and ISO (*n* = 6), respectively for 4 h. F) IL‐1β^+^ neutrophils quantification by flow cytometry of naïve neutrophils treated alone (*n* = 6), with vivo C‐exo (*n* = 6), vivo S‐exo (*n* = 6), and vivo S+P‐exo (*n* = 6), respectively for 4 h. G) Flow cytometry quantification of lung‐infiltrating IL‐1β^+^ neutrophils in 4‐week tumor‐bearing mice in the control group (*n* = 8), chronic stress group (*n* = 8), and chronic stress & propranolol co‐treated group (*n* = 8). H) ELISA analysis of IL‐1β concentrations in the lung supernatant of 4‐week tumor‐bearing mice in the control group (*n* = 24), chronic stress group (*n* = 24), and chronic stress & propranolol co‐treated group (*n* = 24). I) qRT‐PCR analysis of *Nfκβ1*, *Tlr2*, and *Tlr4* expression of naïve neutrophils treated alone (*n* = 3), with vivo C‐exo (*n* = 3), and vivo S‐exo (*n* = 3), respectively for 4 h. J) qRT‐PCR analysis of *Nfκβ1*, *Tlr2*, and *Tlr4* expression of naïve neutrophils treated alone (*n* = 3), with vitro C‐exo (*n* = 3), and vitro ISO‐exo (*n* = 3), respectively for 4 h. K) Flow cytometry quantification of lung‐infiltrating TLR4^+^ neutrophils in 4‐week tumor‐bearing mice of the control group (*n* = 8) and chronic stress group (*n* = 8). L) Quantification of IL‐1β^+^ neutrophils by flow cytometry in naïve neutrophils treated alone (*n* = 5), with vivo C‐exo (*n* = 5), vivo S‐exo (*n* = 5), vivo S‐exo and TLR2 inhibitor (*n* = 5), vivo S‐exo and TLR4 inhibitor (*n* = 5), respectively for 4 h. M) Quantification of IL‐1β^+^ neutrophils by flow cytometry in naïve neutrophils treated alone, with vivo C‐exo (*n* = 6), vitro C‐exo (*n* = 6), vivo C‐exo and a TLR4 agonist (*n* = 6), vitro C‐exo and a TLR4 agonist (*n* = 5), vivo C‐exo and a TLR4 agonist and a NFκβ inhibitor (*n* = 6), and vitro C‐exo and a TLR4 agonist and a NFκβ inhibitor (*n* = 6), respectively for 4 h. The data are shown as mean ± SEM. *: *p* < 0.05, **: *p* < 0.01, ***: *p* < 0.001, and ****: *p* < 0.0001.

To further elucidate the impact of chronic stress‐induced TDEs on neutrophils, we performed qRT‐PCR screening of BM‐PMNs treated alone or with exosomes in different statuses. Among inflammation‐related genes, *Il‐1β* expression was up‐regulated in BM‐PMNs treated with vivo S‐exo or vitro ISO‐exo (**Figure** **4**A,B; Figure S5A,B, Supporting Information). When it came to TDEs in the stress and propranolol co‐treated group (vivo S+P‐exo), this increase was inhibited, indicating that the elevated IL‐1β secretion by neutrophils influenced by chronic stress‐induced TDEs was attributed to the activation of β‐adrenergic receptors on tumor cells (Figure S5C, Supporting Information). This finding was also confirmed by flow cytometry, Western blot (WB), and ELISA assays (Figure 4C–F; Figure S6A–D, Supporting Information). Further, ISO alone could not stimulate neutrophils producing IL‐1β; thus, excluding the direct effects of neuroendocrine factors and emphasizing the indispensable role of TDEs (Figure 4E; Figure S6E,F, Supporting Information).

We next sought to determine whether the polarization of IL‐1β^+^ neutrophils occurred in response to chronic stress in vivo. Neutrophils were the main source of IL‐1β in the lungs of 4T1 tumor‐bearing mice exposed to chronic stress (Figure S6G, Supporting Information). The percentages of pulmonary IL‐1β‐producing neutrophils in the control mice, chronically stressed mice, and mice treated with chronic stress plus propranolol were evaluated, and chronically stressed mice had the highest levels of pulmonary IL‐1β^+^ neutrophils (Figure 4G; Figure S6H, Supporting Information). Similarly, the lung culture supernatants of chronically stressed mice had the highest level of IL‐1β (Figure 4H). We also extracted exosomes from two additional murine breast cancer cell lines (4T07 and EMT6) and found that exosomes from the ISO‐treated group prominently enhanced the neutrophils’ ability to secrete IL‐1β compared to exosomes from the control group (Figure S6I,J, Supporting Information). Together, these data were consistent both in vivo and in vitro and suggested that chronic stress‐induced TDEs polarized IL‐1β^+^ neutrophils.

Next, we aimed to explore the upstream signaling pathway of IL‐1β. qRT‐PCR screening demonstrated that *NF‐κβ*, *Tlr2*, and *Tlr4* were up‐regulated in neutrophils treated with either vivo S‐exo or vitro ISO‐exo and verified by WB (Figure 4C,D,I,J; Figure S5D, Supporting Information). TLRs played a crucial role in innate immunity as they were found on sentinel immune cells, such as neutrophils.^[^ ^16^ ^]^ Compared to control mice, stressed mice exhibited a higher percentage of TLR4^+^ neutrophils; while, the number of TLR2^+^ neutrophils did not change significantly (Figure 4K; Figure S6K, Supporting Information), indicating that TLR4 ligation may be essential for exosomes to mediate their effects on neutrophils. To verify this hypothesis, we utilized TLR2 and TLR4 inhibitors and found that the TLR4 inhibitor markedly abrogated the promotive effects of vivo S‐exo and vitro ISO‐exo (Figure 4L; Figure S6L, Supporting Information). In addition, the NF‐κβ inhibitor also significantly abrogated the promotive effects of vivo S‐exo and vitro ISO‐exo (Figure S6M, Supporting Information). To clarify the interaction between TLR4 and NF‐κβ, BM‐PMNs were treated with exosomes in the presence of agonists or inhibitors for TLR4 and NF‐κβ. We found that the NF‐κβ inhibitor abolished the effects of the TLR4 agonist, indicating that TLR4 was upstream of NF‐κβ (Figure 4M). Using in vitro co‐culture experiments and in vivo mouse models, we demonstrated that chronic stress‐regulated and β‐adrenergic stimulation‐induced TDEs remodeled neutrophils via the TLR4‐NFκβ pathway, which established exosomes as important mediators of immune regulation in the context of cancer.

### 2.5 Chronic Stress Enhances SP1 Accumulation in TDEs

**Figure 5:**
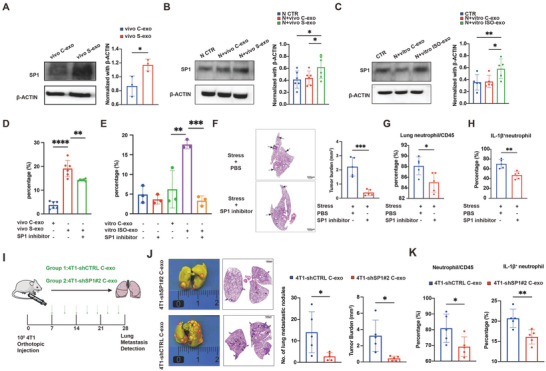
Exosomal SP1 activates the neutrophil TLR4‐NFκβ‐IL‐1β pathway, leading to lung metastasis. A) WB analysis of SP1 expression in vivo C‐exo (*n* = 3) and vivo S‐exo (*n* = 3). B) WB analysis of SP1 expression in BM‐PMNs treated alone (*n* = 6), with vivo C‐exo (*n* = 6), or with vivo S‐exo (*n* = 6) for 4 hours. C) WB analysis of SP1 expression of BM‐PMNs treated alone (*n* = 5), with vitro C‐exo (*n* = 5), and vitro ISO‐exo (*n* = 5), respectively for 4 h. D) Flow cytometry quantification of IL‐1β^+^ neutrophils in BM‐PMNs treated with vivo C‐exo (*n* = 6), vivo S‐exo (*n* = 6), vivo S‐exo and the 500 nm SP1 inhibitor mithramycin A (*n* = 6) for 4 h. E) Flow cytometry quantification of IL‐1β^+^ neutrophils of BM‐PMNs treated alone (*n* = 3), with 500 nm mithramycin A (*n* = 3), vitro C‐exo (*n* = 3), vitro ISO‐exo (*n* = 3), and vitro ISO‐exo and 500 nm mithramycin A (*n* = 3), respectively for 4h. F) Representative H&E staining and quantification of lung metastases in 4‐week tumor‐bearing mice of the chronic stress + PBS injection group (*n* = 5) and the chronic stress + 1 mg kg^−1^ mithramycin A injection group (*n* = 5). Scale bar: 1 mm. G,H) Flow cytometry quantification of lung‐infiltrating neutrophils and lung‐infiltrating IL‐1β^+^ neutrophils in 4‐week tumor‐bearing mice from the chronic stress + PBS injection group (*n* = 5) and the chronic stress + 1 mg kg^−1^ mithramycin A injection group (*n* = 5). I) Diagram illustrating the injection of SP1 control exosomes (4T1‐shCTRL C‐exo) and SP1 knockdown exosomes (4T1‐shSP1#2 C‐exo) into mouse via the tail vein. Exosomes were injected every 3 days into BALB/C mice that had received a subcutaneous injection of 10^5^ 4T1 tumor cells 7 days earlier. J) Representative images of Bouin's and H&E staining for lungs from the 4‐week tumor‐bearing mice injected with 4T1‐shCTRL C‐exo (*n* = 5) or 4T1‐shSP1#2 C‐exo (*n* = 5). Quantification of the number of lung metastatic nodules and burden of lung metastases are shown. K) Flow cytometry quantification of lung‐infiltrating neutrophils and lung‐infiltrating IL‐1β^+^ neutrophils in 4‐week tumor‐bearing mice injected with 4T1‐shCTRL C‐exo (*n* = 5) or 4T1‐shSP1#2 C‐exo (*n* = 5). The data are shown as mean ± SEM. *: *p* < 0.05, **: *p* < 0.01, ***: *p* < 0.001, and ****: *p* < 0.0001.

A growing body of evidence suggests that the transfer of proteins into recipient cells could account for the biological function of exosomes. We next aimed to identify which exosomal factor activated the TLR4 receptor on neutrophils. Transcription factors (TFs) regulate a wide spectrum of cellular functional events, including angiogenesis, cell proliferation, and viability. Previous findings have reported the possibility of transferring TFs to recipient cells via exosomes.^[^ ^17^ ^]^ We found 17 up‐regulated exosomal TFs in vivo S‐exo (Figure S7A,B, Supporting Information), among which four were reported or predicted to be ligands for TLR4, including SP1, NR3C1, STAT1, and SMAD2.^[^ ^18^ ^]^ However, WB analysis showed that only SP1 content was significantly elevated in vivo S‐exo compared with vivo C‐exo (**Figure** **5**A; Figure S7C–E, Supporting Information). Our previous study reported the up‐regulated genes in pulmonary neutrophils of stressed mice.^[^ ^19^ ^]^ DAVID, knock TF, and TRRUST were employed to predict possible upstream TFs of the up‐regulated genes in pulmonary neutrophils of stressed mice, and SP1 was the only intersectional gene (Figure S7F, Supporting Information), which suggested that exosomal SP1 may be involved in the remodeling of neutrophils. In addition, propranolol inhibited the accumulation of SP1 in TDEs (Figure S7G, Supporting Information). In the meantime, vitro ISO‐exo had a higher level of SP1 than vitro C‐exo (Figure S7H, Supporting Information). Treatment with ISO increased the SP1 content in 4T1 cells (Figure S7I, Supporting Information). These data implied that the levels of exosomal SP1 were regulated by the chronic stress‐induced activation of adrenergic β receptors on tumor cells.

We then evaluated the level of SP1 in BM‐PMNs treated with different groups of TDEs and found that BM‐PMNs treated with chronic stress‐induced TDEs demonstrated increased levels of SP1 (Figure 5B,C, Supporting Information), which suggested that stress‐induced TDEs transferred SP1 to neutrophils.

SP1 has been reported to accelerate tumor progression by regulating cell cycle‐related genes to accelerate tumor cell proliferation and facilitating the expression of vascular endothelial growth factors to promote angiogenesis.^[^ ^20^ ^]^ Immunohistochemical staining of SP1 in primary lesions of breast cancer patients demonstrated that patients who developed lung metastasis later showed a higher level of SP1 staining (Figure S7J, Supporting Information).

Collectively, these data implied that the chronic stress‐induced activation of adrenergic β receptors on tumor cells regulated the content of exosomal SP1.

### 2.6 Exosomal SP1 Promotes Lung Metastasis of Breast Cancer by Activating the TLR4‐NFκβ‐IL‐1β Pathway

To assess whether the elevated levels of exosomal SP1 contributed to neutrophil polarization, we exposed BM‐PMNs to exosomes in the presence of the SP1 inhibitor mithramycin A. Mithramycin A significantly abrogated the stimulatory effects of vivo S‐exo and vitro ISO‐exo on the IL‐1β secretion by neutrophils (Figure 5D,E). The prohibitive effect of SP1 inhibitor mithramycin A was also confirmed in 4T1 tumor‐bearing mice exposed to chronic stress. Lung metastasis was impeded when mice were treated with mithramycin A (Figure 5F). The percentages of pulmonary neutrophils and IL‐1β^+^ neutrophils were markedly decreased synchronously (Figure 5G,H; Figure S8A, Supporting Information).

Mithramycin A targets several proto‐oncogenes by binding to their GC‐rich promoters.^[^ ^21^ ^]^ To further address this question, we used SP1 knockdown 4T1 cells (4T1‐shSP1#2) and their controls (4T1‐shCTRL) (Figure S8B, Supporting Information). We extracted exosomes from the supernatants of both cell types treated with either no reagents or ISO. BM‐PMNs were co‐cultured with these exosomes. The promoting effects of exosomes derived from 4T1‐shCTRL treated with ISO (4T1‐shCTRL ISO‐exo) on the up‐regulation of *Il‐1β*, *Nf‐κβ*, *Tlr4*, and *Cxcl2* in neutrophils were largely suppressed in exosomes derived from 4T1‐shSP1#2 treated with ISO (4T1‐shSP1#2 ISO‐exo) (Figure S8C, Supporting Information). The contents of SP1 and TLR4 in neutrophils treated with 4T1‐shSP1#2 ISO‐exo showed no considerable difference when compared to those in neutrophils treated with exosomes derived from 4T1‐shSP1#2 in the control group (4T1‐shSP1#2 C‐exo) (Figure S8D, Supporting Information). 4T1‐shSP1#2 ISO‐exo failed to polarize IL‐1β^+^ neutrophils, unlike 4T1‐shCTRL ISO‐exo (Figure S8E, Supporting Information). Mimicking chronic stress in vitro through β‐adrenergic stimulation on the 4T1‐shSP1#2 limited its exosomes’ ability to function as mediators of immune regulation in cancer.

Next, we aimed to evaluate the implications of exosomal SP1 content. We injected exosomes derived from 4T1‐shCTRL in the control group (4T1‐shCTRL C‐exo) or 4T1‐shSP1#2 C‐exo into 4T1 tumor‐bearing mice (Figure 5I). We found that decreasing the content of exosomal SP1 significantly reduced the lung metastatic burden (Figure 5J). In addition, the number of neutrophils and IL‐1b^+^ neutrophils in the lung tissue markedly decreased (Figure 5K).

In general, these data showed that exosomal SP1 played an indispensable role in activating the TLR4‐NFκβ‐IL‐1β pathway of neutrophils, thereby promoting lung metastasis of breast cancer.

## 3 Discussion

There is a growing awareness that chronic psychological stress is prevalent among cancer patients and is a significant risk factor that can contribute to the progression of tumors. Chronic stress refers to a series of physiological changes brought about by the hypothalamic–pituitary–adrenal axis, sympathetic nervous system activation, and altered neuroendocrine hormones under long‐term stress.^[^ ^22^ ^]^ Previous study has reported that chronic stress favored lung metastasis of breast cancer.^[^ ^23^ ^]^ In our previous study, we reported that chronic stress‐induced pulmonary epithelial cells to produce acetylcholine that enhanced the NETosis of neutrophils and facilitated the capture of cancer cells.^[^ ^19^ ^]^ Nevertheless, we wondered whether chronic stress directly manipulated tumor cells or interfered with their biological behaviors. The components secreted by primary tumors play a crucial role in forming the pre‐metastatic microenvironment in distant tissues. These components include tumor‐derived secretory factors and extracellular vesicles. It has been reported that chronic stress reduced the TGF‐β content in circulating CD63^+^ exosomes in MMTV‐PyMT mice and inhibited tumor growth.^[^ ^24^ ^]^ This finding was inconsistent with the mainstream view on stress and tumor growth, suggesting that chronic stress inhibited tumor growth. Little is known about the molecular profile of chronic stress‐induced TDEs. Therefore, we decided to further investigate these TDEs to determine if they are a significant factor in tumor metastasis

Neutrophils are one of the vital components of the tumor–immune dialogue, and their function remains a subject of debate.^[^ ^25^ ^]^ Our previous research identified a novel group of tumor‐associated aged neutrophils that aggregated in the pre‐metastatic microenvironment, which captured circulating tumor cells by forming mitochondrial‐dependent non‐lethal NETs. This process facilitated the local retention of circulating tumor cells in lung tissue, which can eventually lead to the formation of lung metastases.^[^ ^6b^ ^]^ Hence, we first detected the expression of NET‐related genes in BM‐PMNs co‐cultured with exosomes, which revealed no considerable difference between vivo C‐exo and vivo S‐exo. The results from the immunofluorescence staining of NETs were similar. We also wondered whether chronic stress‐induced TDEs affected the N1/N2 polarization of neutrophils, and we found no notable variations. Inflamed neutrophils constitute the first line of immune defense. Inflammation not only closely relates to the progression of primary tumors but also plays an essential role in promoting distant dissemination.^[^ ^26^ ^]^ Previous studies have shown that lung inflammations resulted in neutrophil recruitment and the production of various inflammatory factors, such as IL‐1β, tumor necrosis factor α, interleukin‐6, and cyclooxygenase 2, leading to eosinophil degranulation and the release of elastase and tissue protease G, which in turn degraded extracellular matrix proteins and destroyed lung homeostasis, thereby promoting lung metastasis.^[^ ^27^ ^]^ IL‐1β has been reported to evoke a systemic inflammatory response, which suppresses the activity of anti‐tumor CD8^+^ T cells and contributes to the establishment of metastases in distant organs.^[^ ^6a^ ^]^ Next, we detected the expression levels of inflammation‐related genes in BM‐PMNs co‐cultured with TDEs. We found that IL‐1β^+^ neutrophils were polarized by chronic stress‐induced TDEs through the TLR4‐NFκβ pathway, thereby contributing to the pro‐metastasis effects mediated by chronic stress.

Among all types of immune cells, pulmonary neutrophils become more abundant as the tumor progresses, emerging as the predominant immune cell type. We speculated that the primary reason exosomes were preferentially taken up by neutrophils was due to their significant abundance. A previous study has reported the potential processes by which red blood cell‐derived microvesicles are taken up by neutrophils, including internalization or fusion of plasma membrane.^[^ ^28^ ^]^ When DiI‐labeled vivo C‐exo and vivo S‐exo were injected into mice, no significant differences were observed in the proportion of DiI^+^ neutrophils among neutrophils between the two groups (Figure S9, Supporting Information). Therefore, we speculated that there may be no specific ligand–receptor interactions between vivo S‐exo and neutrophils, which would make membrane fusion more likely.

SP1 plays a pivotal role in maintaining the expression of many housekeeping genes and participates in various basic activities, such as cell metabolism, proliferation, and death.^[^ ^21^ ^]^ As a TF, SP1 functions primarily by regulating downstream gene expression. However, it can also bind to RNA to influence transcription stability by modulating alternative polyadenylation.^[^ ^29^ ^]^ However, the function of SP1 in exosomes remains elusive. SP1 has been reported to bind to the *Tlr4* promoter, and the CpG demethylation induced by SP1 binding can enhance the transcriptional activity of *Tlr4*, thereby increasing *Tlr4* expression.^[^ ^30^ ^]^ In addition, the TLR4 signaling pathway can also regulate SP1.^[^ ^31^ ^]^ Our study reported that exosomal SP1 provided neutrophils with additional exogenous SP1. After knocking down the *Sp1* gene in the 4T1 cells, we exposed the cells to ISO to simulate chronic stress, and the impact of exosomes on promoting neutrophil secretion of IL‐1β was blocked. Therefore, we have demonstrated the immune regulatory function and metastasis‐promoting mechanism of exosomal SP1 when exposed to chronic stress for the first time.

The feasibility of using exosomes as diagnostic markers or prognostic biomarkers has been demonstrated by a growing body of evidence.^[^ ^32^ ^]^ Moreover, exosomes can serve as drug delivery systems to deliver gene therapy, chemotherapy drugs, and so on.^[^ ^33^ ^]^ Numerous articles indicate that inhibiting exosome secretion through gene editing of exosome‐related genes, such as ras‐related protein rab‐27A (Rab27a) or using GW4869, can effectively restrain tumor progression and enhance the efficacy of immunotherapy;^[^ ^34^ ^]^ yet, there remains a need for considering the safety of these approaches for the host. Imipramine, an anti‐depressant, not only relieves negative emotions in cancer patients but also reduces the release of metastasis‐promoting exosomes.^[^ ^11^ ^]^ We speculate that imipramine may improve the prognosis of breast cancer patients experiencing chronic stress, a hypothesis we will explore in our future studies.

Our study has limitations. Acute stress redistributes leukocytes in mice, including neutrophils, monocytes, B cells, and T cells.^[^ ^35^ ^]^ In a non‐tumor‐bearing background, chronic stress affects lung function and regulates the expression levels of immune‐related genes.^[^ ^36^ ^]^ Further gene editing of Rab27a in the 4T1 cells should be conducted to elucidate the extent to which chronic stress‐induced TDEs remodel the lung immune microenvironment. Another limitation is that, although we demonstrated that patients with lung metastasis exhibit higher expression levels of SP1 in primary lesions, the present study has not identified a correlation between exosomal SP1 and metastasis status in breast cancer patients. These issues will be addressed in subsequent research. In this study, we investigated the mechanism of breast cancer lung metastasis under a specific condition, chronic stress, and elucidated a circuitry involving neuroendocrine changes, exosomes, immunity, and metastasis.

## 4 Conclusion 

**Figure 6:**
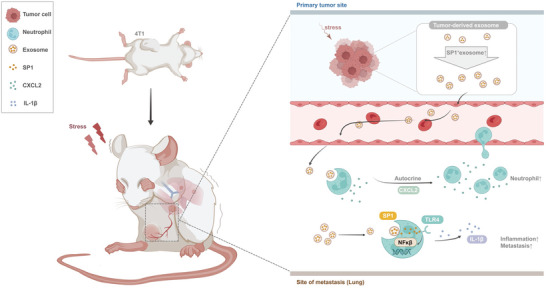
Chronic stress significantly promotes the secretion of TDEs and alters the contents of exosomes by stimulating the adrenergic β receptor. Chronic stress strengthens neutrophil self‐recruitment through the CXCL2 autocrine. Upon exposure to chronic stress, the level of SP1 in TDEs increases, which promotes neutrophil secretion of IL‐1β through the TLR4‐NFκβ pathway, thereby remodeling the lung microenvironment and contributing to the promotion of lung metastasis of breast cancer.

This study is the first to identify the mechanism by which chronic stress fosters lung metastasis of breast cancer by significantly altering TDEs. We demonstrate, for the first time, the novel immune regulatory function of the classical TF, that is, which is mediated by remote transfer through exosomes and affects downstream immune cells. We report that chronic stress remodels the lung immune microenvironment via the SP1‐TLR4‐NFκβ‐IL‐1β axis. We propose a neuroendocrine‐exosome‐immunosuppression‐tumor metastasis axis, which provides new insights into preventing metastasis in breast cancer patients experiencing chronic stress (**Figure** **6**).

## 5 Experimental Section

### Human Samples

Twenty tissue paraffin sections from breast cancer patients were obtained with approval from the Ethics Review Committee of the Second Affiliated Hospital of Zhejiang University School of Medicine (IR2021001330), and all patients provided informed consent. Detailed information on the characteristics of the enrolled patients is provided (Table S1, Supporting Information). The study methodology conformed to the standards set by the Declaration of Helsinki. Eight samples from the no‐metastasis group had a follow‐up period of 10 years without recurrences or distant metastasis.

### Mice

BALB/c mice were purchased from commercial vendors. All mice purchased for use in experiments were female and aged 6–8 weeks. The mice were maintained on a 12‐h dark/light cycle at room temperature. The standard laboratory autoclavable rodent diet was used to feed the mice. All mice were housed in a specific pathogen‐free facility.

BALB/c mice were anesthetized with 0.8% sodium pentobarbital (i.p. injection at a dose of 80 mg kg^−1^) and inoculated with a suspension of 1 × 10^5^ 4T1 or 4T1‐luc cells into the right fourth mammary fat pad to establish the 4T1 tumor‐bearing mouse model.

All animal procedures were approved by the Ethics Review Committee of the Second Affiliated Hospital of Zhejiang University School of Medicine.

### Specimen Acquisition and Processing

Mouse specimens were processed at 9:00 a.m. to minimize the effects of the physiological circadian rhythm. Bone marrow cells were obtained from the tibia and femur, and subsequently, impurities were removed using a 40 µm cell strainer (BD FALCON, #352340). PB was collected from the retro‐orbital vein. The single cell suspensions of primary tumor and lung tissues were obtained by mincing the tissues and digesting them in the RPMI‐1640 medium containing 0.5% fetal bovine serum (FBS), 1 mg mL^−1^ collagenase IV (Sigma–Aldrich), and collagenase I (Sigma–Aldrich).

### Restraint Stress Model

Mice were exposed to restraint stress by being placed in a small device (5 cm × 5 cm × 5 cm) and were subjected to 4–6 h of restraint each day for 28 consecutive days. The holes of the device allowed air to flow. Animals could move their heads and bodies, but were unable to jump or run. Access to food and water was denied to the mice during the restraint period. Once the restraint period ended, mice returned to their home cages and had unrestricted access to food and water. During the restraint period for the chronic stress group, mice in the control group were kept in their home cages without access to food and water. Then, the mice were subjected to open‐field tests and sucrose preference tests to assess the effectiveness of the modeling.

### Cell Culture and Cell Lines

The authenticated murine breast cancer 4T1 and EMT6 cell lines were obtained from the Shanghai Institute of Cell Biology, Chinese Academy of Science. The authenticated murine breast cancer 4T07 cell line was kindly provided by Dr. Lixing Zhan from the Shanghai Institute of Nutrition and Health. All cell lines were authenticated by STR profiling and confirmed to be mycoplasma‐free. All cell lines were cultured at 37 °C in a 5% CO_2_ cell culture incubator with DMEM containing 10% FBS (Gibco) and 1% penicillin–streptomycin solution (Gibco).

### SP1 Knockdown Cell Line

A lentivirus system was used to construct stable 4T1 cells with SP1 knockdown. The SP1 shRNA lentiviral plasmids were purchased from OBiO Technology (Shanghai, China). For lentiviral infection, the viral stock was supplemented with 5 µg mL^−1^ polybrene. Successfully transfected cells were then selected using blasticidin S (Genomeditech). The SP1 shRNA oligonucleotide sequences were as follows:

SP1 shRNA1: CCCTGGAGTAATGCCTAATAT; SP1 shRNA2: CTTCACAACTCAAGCTATTTC; SP1 shRNA3: CCACTCCTTCAGCCCTTATTA; NC shRNA: CCTAAGGTTAAGTCGCCCTCG.

Total protein extraction and WB assays were performed to detect SP1 protein levels. Following verification, the culture medium was replaced with exosome‐free culture medium (DMEM supplemented with 10% exosome‐free FBS). Cells were then allowed to grow for an additional 36–48 h, after which the medium was harvested for exosome isolation.

### Exosome Purification, Characterization, and Quantification

FBS was centrifuged at 110,000 × *g* overnight to remove serum exosomes.

For isolating exosomes from cell lines, cells were cultured in DMEM supplemented with 10% exosome‐free FBS. Supernatants were obtained from cell lines (4T1, 4T07, EMT6) grown to 80–90% confluence for 36–48 h. For isolating exosomes from tissues, murine primary tumors were excised, mechanically separated, and cultured in RPMI‐1640 supplemented with 1% exosome‐free FBS. Supernatants were collected and processed as follows: The supernatants were centrifuged at 300 × *g*, 2000 × *g*, and 10,000 × *g* successively. Subsequently, the supernatant was filtered through a 0.22 µm cell strainer. Then, the supernatant was centrifuged at 110,000 × *g* for 90 min. The pellet was resuspended in PBS and centrifuged again at 110,000 × *g* for 90 min.

The total protein concentration of the exosomes was determined using the BCA Protein Assay kit (Thermo Fisher Scientific).

The morphology of exosomes was examined using Tecnai G2 spirit 120kV transmission electron microscopy (FEI, the U.S.A), as described previously.^[^ ^37^ ^]^

NTA was performed using the NanoSight NS300 system (Malvern Panalytical, U.K.) to examine the size distribution and concentration of the exosomes. Each sample was resuspended in particle‐free PBS and analyzed three times.

### WB

Cells and exosomes were isolated and lyzed using pre‐cooled lysis buffer supplemented with a cocktail of protease and phosphatase inhibitors (Thermo Fisher). Protein concentrations were measured using a BCA assay kit. Total protein was separated by SDS‐PAGE and transferred onto polyvinylidene difluoride membranes (Bio‐rad). After blocking with Protein Free Rapid Blocking Buffer (Epizyme Biotech), the membranes were incubated overnight at 4 °C with the indicated primary antibodies: anti‐CD63, anti‐Tumor Susceptibility 101 (TSG101), anti‐ALG‐2‐Interacting Protein X (ALIX), anti‐Calnexin, anti‐Actin Beta (β‐ACTIN), anti‐Glyceraldehyde‐3‐Phosphate Dehydrogenase (GAPDH), anti‐IL‐1β, anti‐NFκβ p65 (phospho S536), and anti‐NFκβ p65. This was followed by incubation with secondary antibodies and visualization using enhanced chemiluminescence reagent (Bio‐rad). Antibody binding was visualized with Azure 300 (Azure Biosystems) and ImageQuant 800 (AMERSHAM) and analyzed by ImageJ software.

### In Vivo Tumor Metastasis Model

4T1‐luc (1 × 10^4^ cells in 100 µL PBS) was injected into BALB/c mice via the tail vein. The mice were then randomly assigned to two groups. One group received 200 µL PBS containing DMSO intraperitoneally daily before restraint treatment and was designated as the stress + PBS injection group. The other group received 1.25 mg kg^−1^ GW4869 (Selleck) in 200 µL PBS intraperitoneally daily before restraint treatment and was designated as the stress + GW4869 injection group. In vivo imaging was conducted using D‐Luciferin (MedChemExpress) and the IVIS Spectrum (PerkinElmer).

### Lung Histology

Tumor nodes on the lung were counted via Bouin's solution staining using double blind method. Lungs were fixed in 3.7% formaldehyde/PBS (pH 7.4), dehydrated, embedded in paraffin, and sectioned. H&E staining was performed. The slides were then scanned using a Pannoramic MIDI instrument (3DHISTECH Ltd.). Tumor burden was evaluated using ImageJ software by calculating the area of the lung metastases in the presented figures.

### Immunohistochemistry (IHC) Staining

IHC was performed using a standard protocol according to the manufacturer's instructions (Absin). Primary antibodies specific for SP1 (Proteintech), ADRB1 (Proteintech), ADRB2 (Proteintech), and Cytokeratin 8 (Servicebio) and HRP‐labeled secondary antibodies specific for rabbit IgG (Servicebio) were used. The slides were then scanned using a Pannoramic MIDI instrument (3DHISTECH Ltd.). At least five random fields per sample were imaged as TIFF files. The IHC staining score was semi‐quantitatively evaluated by determining the percentage of positive staining area using ImageJ software. The IHC staining was confirmed by two researchers (Leyi Zhang and Jini Yang), who were blinded to the clinicopathological characteristics.

### Flow Cytometry Analysis

The dissociated single cells from murine tissues were suspended in 100 µL of cell staining buffer and stained with Zombie Red or 7‐aminoactinomycin D to distinguish between live and dead cells, as per the manufacturer's instructions. They were stained with a series of fluorescence activated cell sorting (FACS) antibodies, including CD45, CD3, CD4, CD8a, T cell receptor (TCR) γ/δ, CD11 antigen‐like family member B (CD11B), lymphocyte antigen 6 family member G (LY6G), lymphocyte antigen 6 family member C (LY6C), CD284, and CD282 (Biolegend). For intracellular staining, cells were fixed and permeabilized using the fixation and permeabilization solution (BD Biosciences). Subsequently, the cells were stained with anti‐IL‐1β antibody (Thermo fisher). After staining, the cells were analyzed on a FACS Canto II flow cytometer (BD Biosciences). Data were analyzed using FlowJo software Version 10.0 for Mac.

### Magnetic Isolation of Neutrophils

Neutrophils were isolated using a mouse neutrophil isolation kit (Miltenyi) according to the manufacturer's instructions. An LS column and Midi‐MACS separator (Miltenyi) were used for subsequent magnetic sorting. The enriched cell suspension was collected into a new tube and was ready for further in vitro experiments.

### In Vitro Co‐Culture of Neutrophils with TDEs

BM‐PMNs were obtained from the bone marrow of 4T1 tumor‐bearing mice at 2 weeks post‐inoculation. To analyze IL‐1β secretion from neutrophils, 10,000 neutrophils and 10 µg exosome in exosome‐free RPMI‐1640 medium were plated into U‐bottom 96‐well plates.

Reagents involved in the co‐culture were as follows: O‐Vanillin (100 µm), Resatorvid (10 µm), LPS (100 ng mL^−1^), and BAY‐11‐7082 (1 µm).

Following a 4‐h treatment, neutrophils were detected for IL‐1β secretion by flow cytometry. For qRT‐PCR, WB, ELISA, and Transwell assays, 1 × 10^6^ neutrophils were treated with 50 µg exosome and plated into 6‐well plates for 4 h. The supernatants were collected and used for ELISA and Transwell assays, and the cells were harvested for protein extraction and WB analysis.

Pulmonary neutrophils were isolated from the lungs of 4T1 tumor‐bearing mice 2 weeks after inoculation. Neutrophils (1 × 10^6^) labeled with DiI were co‐cultured with 50 µg of TDEs for 4h. The co‐cultured cells were then harvested and stained with Hoechst (Thermo Fisher). Images were captured at 40× magnification using an Olympus FV3000 confocal microscope and processed with Olympus FV31 software.

### qRT‐PCR

Mouse tumor tissues were ground in liquid nitrogen; while, cell samples were directly added to TRlzol reagent (Invitrogen) for RNA extraction. RNA concentration was quantified using a NanoDrop OncC spectrophotometer (Thermo Fisher). cDNA templates were synthesized using the HiScript II 1st Strand cDNA Synthesis Kit (Vazyme). qRT‐PCR was conducted using the ChamQ Universal SYBR qPCR Master Mix (Vazyme) on a 7500 Fast Real‐Time System (Applied Biosystems). Refer to Table S2, Supporting Information for the primer sequences used.

### Treatment of BALB/C Mice with TDEs

As previously described, BALB/C mice were inoculated with 4T1 cells. One week after modeling, the mice were intravenously administrated with either 200 µL of PBS or 20 µg of exosomes in 200 µL of PBS, once every 2 days. Following 3 weeks of intervention, the mice were sacrificed.

For exosome tracking, exosomes (40 µg per mouse) were labeled with DiI (YEASEN) and injected into BALB/C mice via the tail vein. 4 h post‐injection, the PB and lungs were harvested to detect fluorescence signals by flow cytometry.

For exosome in vivo biodistribution analysis, exosomes (100 µg per mouse) were labeled with DiR (YEASEN) and injected into BALB/C mice via the tail vein. 4 h and 24 h post‐injection, organs were harvested to detect fluorescence signals.

### In Vivo β‐Adrenergic Receptor Stimulation and Blockade

BALB/C mice were orthotopically injected with 4T1 cells, as mentioned above, and then randomly divided into two groups. For ISO administration, the mice were injected intraperitoneally with 10 mg kg^−1^ ISO in 200 µL of PBS daily. For propranolol administration, the mice were injected intraperitoneally with 2 mg kg^−1^ propranolol in 200 µL of PBS daily. Four weeks after tumor implantation, the mice were sacrificed, and their lungs were harvested.

### Cytokine and ELISA Measurement

The supernatant was isolated from the culture conditions, centrifuged at 3000 rpm for 5 min to remove cellular debris, and then transferred to a new Eppendorf tube for storage at −80 °C until use.

ELISA kits for IL‐1β (Biolegend), CCL3, and CXCL2 (Multi sciences) were used according to the manufacturer's instructions.

### Immunofluorescence Staining

To detect exosome uptake in neutrophils, lungs were harvested and 4–5 µm paraffin‐embedded sections were obtained, followed by deparaffinization and rehydration. Antigenic retrieval was performed using a Tris‐EDTA (pH = 9) buffer in a thermostatted bath at 98 °C for 25 min. The sections were then incubated in a 3% hydrogen peroxide at room temperature in darkness for 25 min, followed by blocking with 3% bovine serum albumin. Primary antibodies staining was conducted overnight at 4 °C in a humidified staining container with anti‐MPO (Abcam). The sections were then washed with PBS and stained with secondary rabbit IgG (Abcam) for 1 h at room temperature. The slides were then scanned using a Pannoramic MIDI instrument (3DHISTECH Ltd.).

### Neutrophils Depletion In Vivo

The anti‐mouse Ly‐6G antibody (Selleck, #A2158) was administered at a dose of 1.5 mg kg^−1^ via the tail vein preceding the exosome injection, as indicated in the schematic diagram. PBS was administered at an equivalent volume as a control.

### Neutrophil Chemotaxis Assay

BM‐PMNs were isolated and resuspended in serum‐free culture medium, and seeded into 24‐well Transwell inserts (5.0 mm, Corning). Complete medium 1:1, supplemented with the supernatants of neutrophils treated with no additional reagents (control), vivo C‐exo, vivo S‐exo, vitro C‐exo, vitro ISO‐exo, or CXCL2 neutralizing antibody (2 µg mL^−1^, R&D Systems), was added to the remaining receiver wells. After 1–2 h, the cells in the receiver wells were collected for flow cytometry.

### Proteomic Analysis

Briefly, 200 µg of protein from vivo C‐exo and vivo S‐exo was processed using the filter‐aided sample preparation procedure. A 100‐µg peptide mixture from each sample was labeled using TMT reagent according to the manufacturer's instructions (Thermo Scientific). The labeled peptides were then fractionated using the High pH reversed‐phase peptide fractionation kit (Thermo Scientific). LC‐MS/MS analysis was performed on a Q Exactive mass spectrometer (Thermo Scientific) coupled to an Easy nLC system (Proxeon Biosystems, now Thermo Fisher Scientific). The MS raw data for each sample were searched for using the MASCOT engine (Matrix Science) incorporated into Proteome Discoverer 1.4 software for identification and quantification analysis.

### Bioinformatics Analyses

The Venn diagram was used to identify overlaps between the up‐regulated proteins of vivo S‐exo in proteome analysis and the list of mouse TFs.

Gene ontology (GO) enrichment analysis was conducted using the ‘clusterProfiler’ and ‘ccgraph’ packages in R.

PPI analysis was conducted using the STRING database (http://string‐db.org/). The results were downloaded in XGMML format and imported into Cytoscape software (http://www.cytoscape.org/, version 3.2.1) for visualization and subsequent analysis of functional PPI networks. In addition, the protein degree was calculated to evaluate the importance of each protein within the PPI network.

The up‐regulated genes of pulmonary neutrophils from stressed mice (Table S3, Supporting Information) were used to predict exosomal key TFs using DAVID (https://david.ncifcrf.gov/),^[^ ^38^ ^]^ knockTF (http://www.licpathway.net/KnockTF/index.html),^[^ ^39^ ^]^ and TRRUST (www.grnpedia.org/trrust) analysis (Table S4, Supporting Information).^[^ ^40^ ^]^ The Venn diagram was also used to identify overlaps between the predicted TFs from DAVID, knock TF, and TRRUST.

Predictions of TLR4 ligands were conducted using the STRING database (http://string‐db.org/), cellinker (http://www.rna‐society.org/cellinker/),^[^ ^18a^ ^]^ and CellTalkDB (http://tcm.zju.edu.cn/celltalkdb).^[^ ^18b^ ^]^

### Differentially Expressed Genes in GSE154685

They (https://www.ncbi.nlm.nih.gov/geo/query/acc.cgi?acc=GSE154685) were identified using the ‘LIMMA’ package in R. Tumors were removed from 4T1 tumor‐bearing mice that had been subjected to chronic restraint stress for 2 h daily over consecutive 28 days, which were designated as the stress group. Tumors removed from 4T1 tumor‐bearing mice without any treatment served as the control group. Genes with a *p*‐value less than 0.05 were considered differentially expressed between the stress and control groups. A total of 2786 differentially expressed mRNAs were identified (Table S5, Supporting Information).

### Statistical Analysis

All statistical analyses were conducted using GraphPad Prism (version 9.0, https://www.graphpad‐prism.cn/) and R software (version 4.0.0, http://www.Rproject.org). Data are presented as the mean ± standard deviation. Paired and unpaired two‐tailed Student's *t*‐test, as well as Mann–Whitney U‐tests, were used for comparisons between two groups. One‐way ANOVA or two‐way ANOVA followed by Bonferroni's test was used for multiple comparisons.

A value of *p* < 0.05 (two‐sided) was considered statistically significant.

Three or more biological replicates were used for each study. Each dot represents one biological replicate. Details of the statistical analysis are provided in the figure legends. All in vitro experiments were conducted with at least three independent repeats.

## Conflict of Interest

The authors declare no conflict of interest.

## Authors’ Contributions

L.Z., J.P., and M.W. contributed equally to this study. L.Z. and J.H. initiated and organized the study. L.Z. performed the majority of the experiments, conducted the statistical analysis, and drafted the manuscript. J.P., M.W., and J.Y. participated in the animal experiments and in vitro experiments. S.Z., L.L., X.H., Z.W., L.P., P.L., F.J., G.R., Y.Z., and D.X. assisted and performed parts of the experiments. S.Z., F.Q., and J.H. edited the manuscript. All authors contributed to the article and approved the submitted version.

## Supporting information



Supporting Information

Supporting Information

Supporting Information

Supporting Information

Supporting Information

Supporting Information

## Data Availability

The proteomics data have been deposited to the ProteomeXchange Consortium (http://proteomecentral.proteomexchange.org) via the iProX partner repository with the dataset identifier PXD048125. The dataset analyzed for this study is openly available in the Gene Expression Omnibus (GEO) Dataset (https://www.ncbi.nlm.nih.gov/geo/query/acc.cgi?acc=GSE154685) with the accession number GSE154685. Data supporting the findings of this manuscript are available from the corresponding author upon request.

## References

[1] a) J. Zhou , Z. Liu , L. Zhang , X. Hu , Z. Wang , H. Ni , Y. Wang , J. Qin , Cancer Res. Treat. 2020, 52, 830; 32138468 10.4143/crt.2019.510 PMC7373858

[2] A. Valachis , P. Carlqvist , Y. Ma , M. Szilcz , J. Freilich , S. Vertuani , B. Holm , H. Lindman , Br. J. Cancer 2022, 127, 720. 35597870 10.1038/s41416-022-01845-z PMC9381497

[3] Z. Zhao , L. Fang , P. Xiao , X. Sun , L. Zhou , X. Liu , J. Wang , G. Wang , H. Cao , P. Zhang , Y. Jiang , D. Wang , Y. Li , Adv. Mater. 2022, 34, 2205462. 10.1002/adma.202205462 35759925

[4] a) M. Pein , J. Insua‐Rodríguez , T. Hongu , A. Riedel , J. Meier , L. Wiedmann , K. Decker , M. A. G. Essers , H. P. Sinn , S. Spaich , M. Sütterlin , A. Schneeweiss , A. Trumpp , T. Oskarsson , Nat. Commun. 2020, 11, 1494; 32198421 10.1038/s41467-020-15188-x PMC7083860

[5] B. Medeiros , A. Allan , Int. J. Mol. Sci. 2019, 20, 2272.31071959

[6] a) S. B. Coffelt , K. Kersten , C. W. Doornebal , J. Weiden , K. Vrijland , C. S. Hau , N. J. M. Verstegen , M. Ciampricotti , L. Hawinkels , J. Jonkers , K. E. de Visser , Nature 2015, 522, 345; 25822788 10.1038/nature14282 PMC4475637

[7] T. Heidt , H. B. Sager , G. Courties , P. Dutta , Y. Iwamoto , A. Zaltsman , C. von Zur Muhlen , C. Bode , G. L. Fricchione , J. Denninger , C. P. Lin , C. Vinegoni , P. Libby , F. K. Swirski , R. Weissleder , M. Nahrendorf , Nat. Med. 2014, 20, 754. 24952646 10.1038/nm.3589 PMC4087061

[8] a) L. Zhang , B. Zheng , Y. Bai , J. Zhou , X. H. Zhang , Y. Q. Yang , J. Yu , H. Y. Zhao , D. Ma , H. Wu , J. K. Wen , Adv. Sci. 2023, 10, e2300560; 10.1002/advs.202300560 PMC10558653 37590310

[9] E. A. Makrygianni , G. P. Chrousos , Neuroendocrinology 2023, 113, 120. 36137504 10.1159/000527182

[10] B. Cui , Y. Luo , P. Tian , F. Peng , J. Lu , Y. Yang , Q. Su , B. Liu , J. Yu , X. Luo , L. Yin , W. Cheng , F. An , B. He , D. Liang , S. Wu , P. Chu , L. Song , X. Liu , H. Luo , J. Xu , Y. Pan , Y. Wang , D. Li , P. Huang , Q. Yang , L. Zhang , B. P. Zhou , S. Liu , G. Xu , et al., J. Clin. Invest. 2019, 129, 1030. 30688660 10.1172/JCI121685 PMC6391112

[11] M. Catalano , L. O'Driscoll , J. Extracell. Vesicles 2020, 9, 1703244. 32002167 10.1080/20013078.2019.1703244 PMC6968539

[12] a) P. H. Thaker , L. Y. Han , A. A. Kamat , J. M. Arevalo , R. Takahashi , C. Lu , N. B. Jennings , G. Armaiz‐Pena , J. A. Bankson , M. Ravoori , W. M. Merritt , Y. G. Lin , L. S. Mangala , T. J. Kim , R. L. Coleman , C. N. Landen , Y. Li , E. Felix , A. M. Sanguino , R. A. Newman , M. Lloyd , D. M. Gershenson , V. Kundra , G. Lopez‐Berestein , S. K. Lutgendorf , S. W. Cole , A. K. Sood , Nat. Med. 2006, 12, 939; 16862152 10.1038/nm1447

[13] a) J. Wang , E. E. Bonacquisti , A. D. Brown , J. Nguyen , Cells 2020, 9, e0028752272; 10.3390/cells9030660 PMC7140620 32182815

[14] Y. Ma , G. Kroemer , Nat. Rev. Immunol. 2023, 24 ,264. 37833492 10.1038/s41577-023-00949-8

[15] D. S. Vinay , E. P. Ryan , G. Pawelec , W. H. Talib , J. Stagg , E. Elkord , T. Lichtor , W. K. Decker , R. L. Whelan , H. Kumara , E. Signori , K. Honoki , A. G. Georgakilas , A. Amin , W. G. Helferich , C. S. Boosani , G. Guha , M. R. Ciriolo , S. Chen , S. I. Mohammed , A. S. Azmi , W. N. Keith , A. Bilsland , D. Bhakta , D. Halicka , H. Fujii , K. Aquilano , S. S. Ashraf , S. Nowsheen , X. Yang , et al., Semin. Cancer Biol. 2015, 35, S185. 25818339 10.1016/j.semcancer.2015.03.004

[16] S. N. Lester , K. Li , J. Mol. Biol. 2014, 426, 1246. 24316048 10.1016/j.jmb.2013.11.024 PMC3943763

[17] a) Q. Wang , L. Zhang , Z. Sun , B. Chi , A. Zou , L. Mao , X. Xiong , J. Jiang , L. Sun , W. Zhu , Y. Ji , Mater. Today Bio 2021, 12, 100171; 10.1016/j.mtbio.2021.100171 PMC8640519 34901821

[18] a) Y. Zhang , T. Liu , J. Wang , B. Zou , L. Li , L. Yao , K. Chen , L. Ning , B. Wu , X. Zhao , D. Wang , Bioinformatics 2021, 37, 2025; 10.1093/bioinformatics/btab036 PMC7929259 33471060

[19] J. Pan , L. Zhang , X. Wang , L. Li , C. Yang , Z. Wang , K. Su , X. Hu , Y. Zhang , G. Ren , J. Jiang , P. Li , J. Huang , J. Exp. Clin. Cancer Res. 2023, 42, 255. 37773152 10.1186/s13046-023-02836-5 PMC10540414

[20] K. Beishline , J. Azizkhan‐Clifford , FEBS J. 2015, 282, 224. 25393971 10.1111/febs.13148

[21] J. Kormanec , R. Novakova , D. Csolleiova , L. Feckova , B. Rezuchova , B. Sevcikova , D. Homerova , Appl. Microbiol. Biotechnol. 2020, 104, 7701. 32686008 10.1007/s00253-020-10782-x

[22] Y. M. Ulrich‐Lai , J. P. Herman , Nat. Rev. Neurosci. 2009, 10, 397. 19469025 10.1038/nrn2647 PMC4240627

[23] a) H. Chen , D. Liu , L. Guo , X. Cheng , N. Guo , M. Shi , J. Pathol. 2018, 244, 49; 28940209 10.1002/path.4988

[24] R. P. Dawes , K. A. Burke , D. K. Byun , Z. Xu , P. Stastka , L. Chan , E. B. Brown , K. S. Madden , Breast Cancer 2020, 14, 1178223420931511. 32595275 10.1177/1178223420931511 PMC7301655

[25] B. M. Szczerba , F. Castro‐Giner , M. Vetter , I. Krol , S. Gkountela , J. Landin , M. C. Scheidmann , C. Donato , R. Scherrer , J. Singer , C. Beisel , C. Kurzeder , V. Heinzelmann‐Schwarz , C. Rochlitz , W. P. Weber , N. Beerenwinkel , N. Aceto , Nature 2019, 566, 553. 30728496 10.1038/s41586-019-0915-y

[26] E. Elinav , R. Nowarski , C. A. Thaiss , B. Hu , C. Jin , R. A. Flavell , Nat. Rev. Cancer 2013, 13, 759. 24154716 10.1038/nrc3611

[27] a) T. El Rayes , R. Catena , S. Lee , M. Stawowczyk , N. Joshi , C. Fischbach , C. A. Powell , A. J. Dannenberg , N. K. Altorki , D. Gao , V. Mittal , Proc. Natl. Acad. Sci. U. S. A. 2015, 112, 16000; 26668367 10.1073/pnas.1507294112 PMC4703007

[28] G. P. de Oliveira , J. A. Welsh , B. Pinckney , C. C. Palu , S. Lu , A. Zimmerman , R. H. Barbosa , P. Sahu , M. Noshin , S. Gummuluru , J. Tigges , J. C. Jones , A. R. Ivanov , I. C. Ghiran , J. Extracell. Biol. 2023, 11, e107. 10.1002/jex2.107 PMC10629908 37942280

[29] J. Song , S. Nabeel‐Shah , S. Pu , H. Lee , U. Braunschweig , Z. Ni , N. Ahmed , E. Marcon , G. Zhong , D. Ray , K. C. H. Ha , X. Guo , Z. Zhang , T. R. Hughes , B. J. Blencowe , J. F. Greenblatt , Mol. Cell 2022, 82, 3135. 35914531 10.1016/j.molcel.2022.06.031

[30] a) T. W. Kim , S. J. Lee , B. M. Oh , H. Lee , T. G. Uhm , J. K. Min , Y. J. Park , S. R. Yoon , B. Y. Kim , J. W. Kim , Y. K. Choe , H. G. Lee , Oncotarget 2016, 7, 4195; 26675260 10.18632/oncotarget.6549 PMC4826199

[31] a) S. J. Lee , K. W. Seo , C. D. Kim , Korean J. Physiol. Pharmacol. 2015, 19, 263; 25954132 10.4196/kjpp.2015.19.3.263 PMC4422967

[32] a) X. X. Peng , R. Yu , X. Wu , S. Y. Wu , C. Pi , Z. H. Chen , X. C. Zhang , C. Y. Gao , Y. W. Shao , L. Liu , Y. L. Wu , Q. Zhou , J. Immunother. Cancer 2020, 8, e000376; 31959728 10.1136/jitc-2019-000376 PMC7057418

[33] a) M. Yu , C. Gai , Z. Li , D. Ding , J. Zheng , W. Zhang , S. Lv , W. Li , Cancer Sci. 2019, 110, 3173; 31464035 10.1111/cas.14181 PMC6778638

[34] S. M. Morrissey , F. Zhang , C. Ding , D. E. Montoya‐Durango , X. Hu , C. Yang , Z. Wang , F. Yuan , M. Fox , H. G. Zhang , H. Guo , D. Tieri , M. Kong , C. T. Watson , R. A. Mitchell , X. Zhang , K. M. McMasters , J. Huang , J. Yan , Cell Metab. 2021, 33, 2040. 34559989 10.1016/j.cmet.2021.09.002 PMC8506837

[35] W. C. Poller , J. Downey , A. A. Mooslechner , N. Khan , L. Li , C. T. Chan , C. S. McAlpine , C. Xu , F. Kahles , S. He , H. Janssen , J. E. Mindur , S. Singh , M. G. Kiss , L. Alonso‐Herranz , Y. Iwamoto , R. H. Kohler , L. P. Wong , K. Chetal , S. J. Russo , R. I. Sadreyev , R. Weissleder , M. Nahrendorf , P. S. Frenette , M. Divangahi , F. K. Swirski , Nature 2022, 607, 578. 35636458 10.1038/s41586-022-04890-z PMC9798885

[36] M. Barnthouse , B. L. Jones , Clin. Rev. Allergy Immunol. 2019, 57, 427. 31079340 10.1007/s12016-019-08736-x

[37] P. F. Zhang , C. Gao , X. Y. Huang , J. C. Lu , X. J. Guo , G. M. Shi , J. B. Cai , A. W. Ke , Mol. Cancer 2020, 19, 110. 32593303 10.1186/s12943-020-01222-5 PMC7320583

[38] a) D. W. Huang , B. T. Sherman , Q. Tan , J. Kir , D. Liu , D. Bryant , Y. Guo , R. Stephens , M. W. Baseler , H. C. Lane , R. A. Lempicki , Nucleic Acids Res. 2007, 35, W169; 17576678 10.1093/nar/gkm415 PMC1933169

[39] C. Feng , C. Song , Y. Liu , F. Qian , Y. Gao , Z. Ning , Q. Wang , Y. Jiang , Y. Li , M. Li , J. Chen , J. Zhang , C. Li , Nucleic Acids Res. 2020, 48, D93. 31598675 10.1093/nar/gkz881 PMC6943067

[40] a) H. Han , H. Shim , D. Shin , J. E. Shim , Y. Ko , J. Shin , H. Kim , A. Cho , E. Kim , T. Lee , H. Kim , K. Kim , S. Yang , D. Bae , A. Yun , S. Kim , C. Y. Kim , H. J. Cho , B. Kang , S. Shin , I. Lee , Sci. Rep. 2015, 5, 11432; 26066708 10.1038/srep11432 PMC4464350

