# Supplementary material for: Chronic Stress‐Induced and Tumor Derived SP1+ Exosomes Polarizing IL‐1β+ Neutrophils to Increase Lung Metastasis of Breast Cancer
Source: Adv Sci (Weinh). 2024 Dec 4;12(4):2310266. doi: 10.1002/advs.202310266 (PMC11789585; doi:10.1002/advs.202310266)
Supplement: Supplementary file 1 — Supporting Information [file ADVS-12-2310266-s002.docx]

Supporting Information

**Chronic Stress-Induced and Tumor Derived SP1^+^ Exosomes Polarizing IL-1β^+^ Neutrophils to Increase Lung Metastasis of Breast Cancer**

*Leyi Zhang, Jun Pan, Meijun Wang, Jini Yang, Sangsang Zhu, Lili Li, Xiaoxiao Hu, Zhen Wang, Liwei Pang, Peng Li, Fang Jia, Guohong Ren, Yi Zhang, Danying Xu, Fuming Qiu, and Jian Huang**


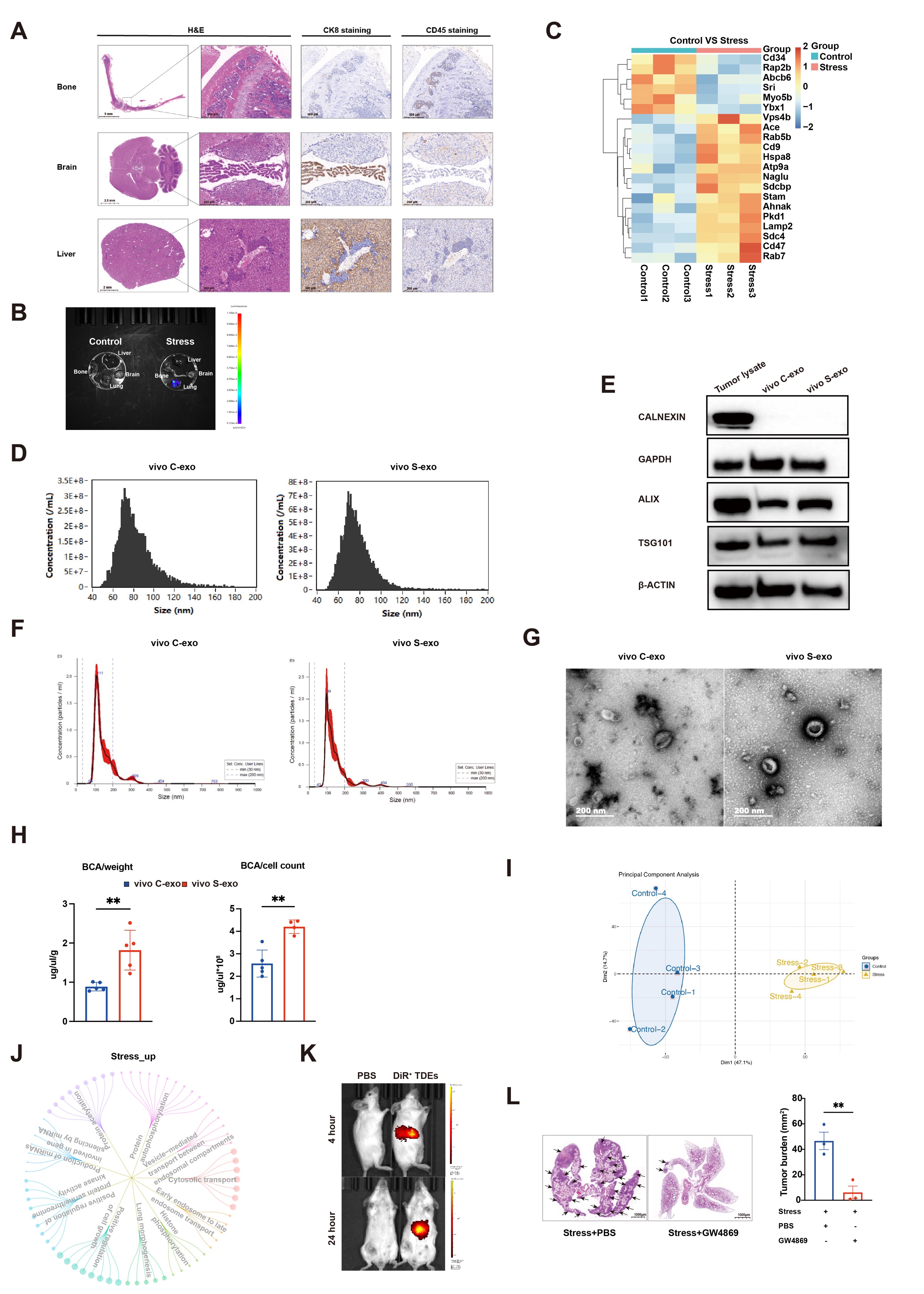


**Figure S1.**

**A**. Representative H&E staining, cytokeratin-8 (CK8) and CD45 immunohistochemical staining of the bone, brain and liver tissues from 4-week tumor-bearing mice in the chronic stress group. **B**. Representative bioluminescent image of the lung, liver, bone, and brain of mice injected with luciferase-expressing 4T1 tumor cells orthotopically in the control group and the stress group, four weeks post-injection. **C.** Heatmap of differentially expressed exosome-related genes in GSE154685 dataset (p<0.05). **D.** The representative images of nanoflow cytometry analysis of extracted exosomes. **E.** The representative images of WB analysis showing the expression of negative marker (CALNEXIN), positive markers (ALIX, TSG101), and reference markers (GAPDH, β-ACTIN) in extracted exosomes. **F.** The representative images of NTA of extracted exosomes. **G**. The representative transmission electron microscopy images of extracted exosomes. **H**. The concentrations of vivo C-exo and vivo S-exo were measured using the BCA method and calibrated with the weight of the original tumor tissues or the cell counts of digested tumor tissues (n=4-5/per group). **I**. Principal component analysis of the proteome analysis of vivo C-exo (n=4) and vivo S-exo (n=4) is shown. **J**. Gene ontology analysis of up-regulated proteins of the proteome analysis of vivo S-exo. **K.** *In vivo* biodistribution of 100 µg DiR-labeled TDEs injected into 4T1 tumor-bearing BALB/c mice via tail vein at 4 and 24 hours. **L**. Representative H&E staining and quantification of lung metastases in 4-week tumor-bearing mice injected with luciferase-expressing 4T1 tumor cells via the tail vein in the stress + PBS group (n=3) and the stress + GW4869 group (n=3). Scale bar, 1 mm. The data are shown as mean ± SEM. **: p < 0.01.


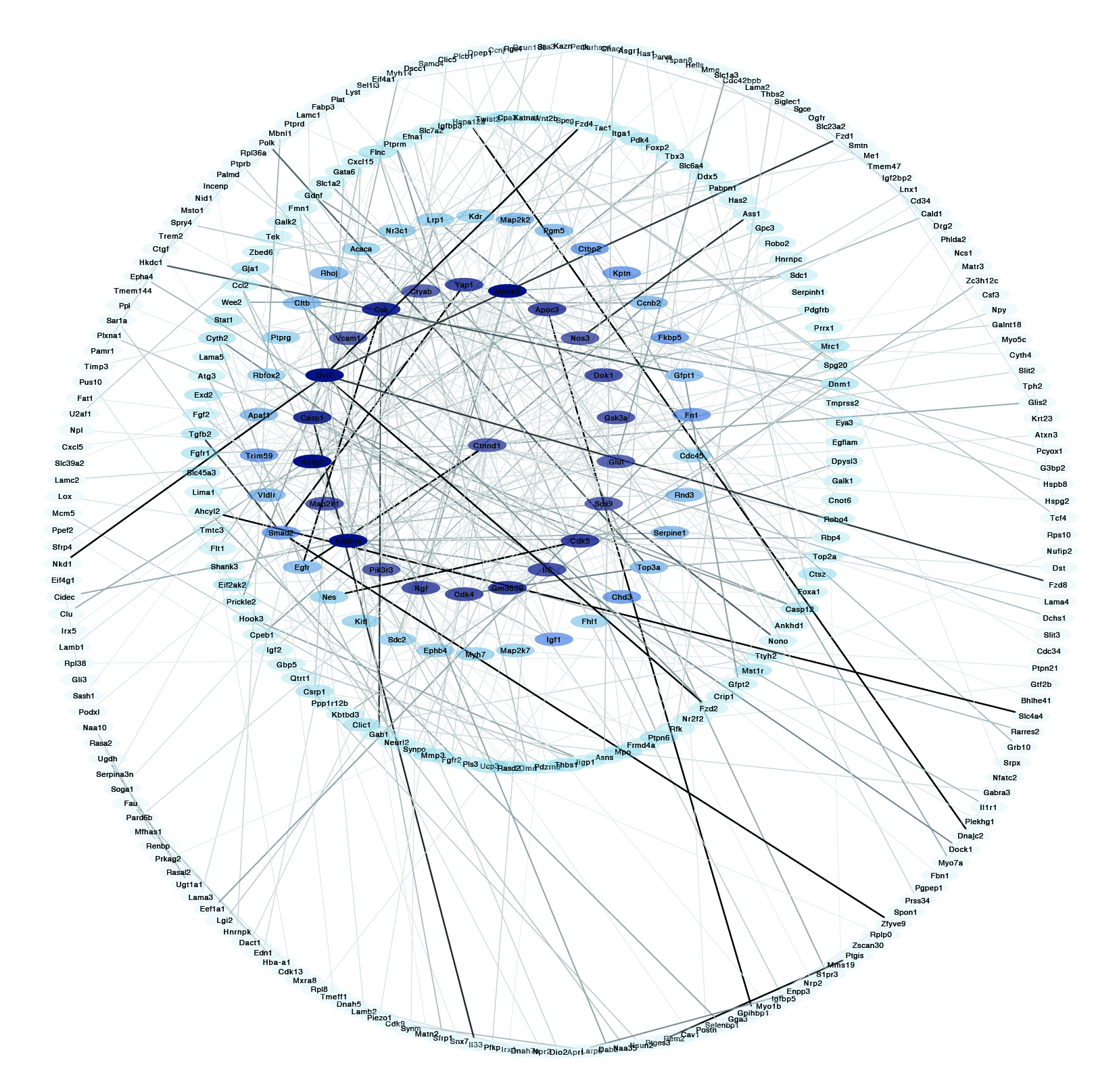


**Figure S2.**

PPI analysis of up-regulated proteins of vivo S-exo in proteome analysis using STRING database.


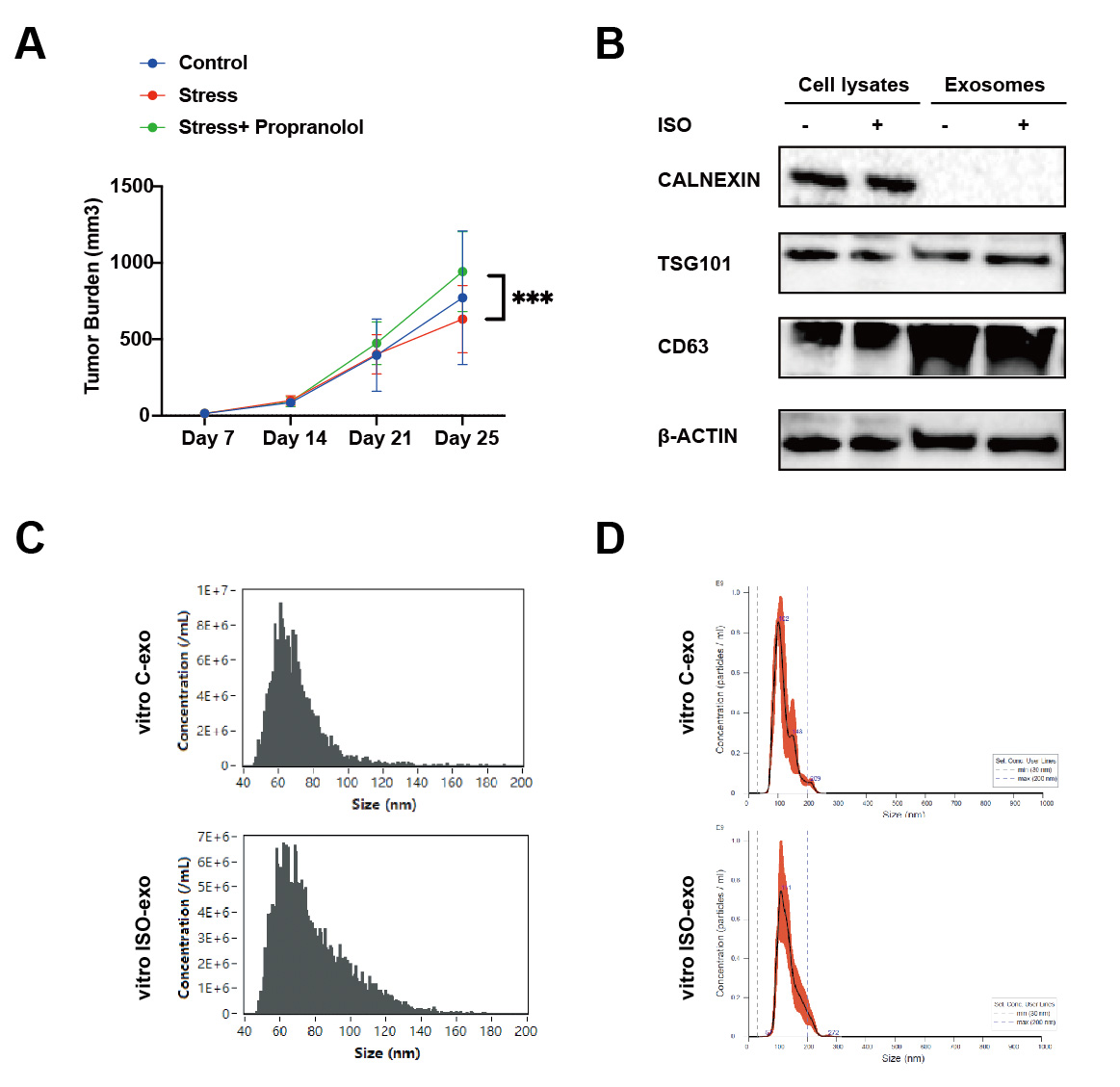


**Figure S3.**

**A.** The growth curve of the primary tumors in the control group, chronic stress group, and chronic stress & propranolol co-treated group (n=11 for control, n=10 for stress and stress & propranolol co-treated groups). **B**. WB analysis showing the expression of negative marker (CALNEXIN), positive markers (TSG101, CD63), and reference marker (β-ACTIN) in extracted exosomes and 4T1 cell lysates. **C**. The representative images of nanoflow analysis of extracted exosomes. **D**. The representative images of NTA of extracted exosomes.


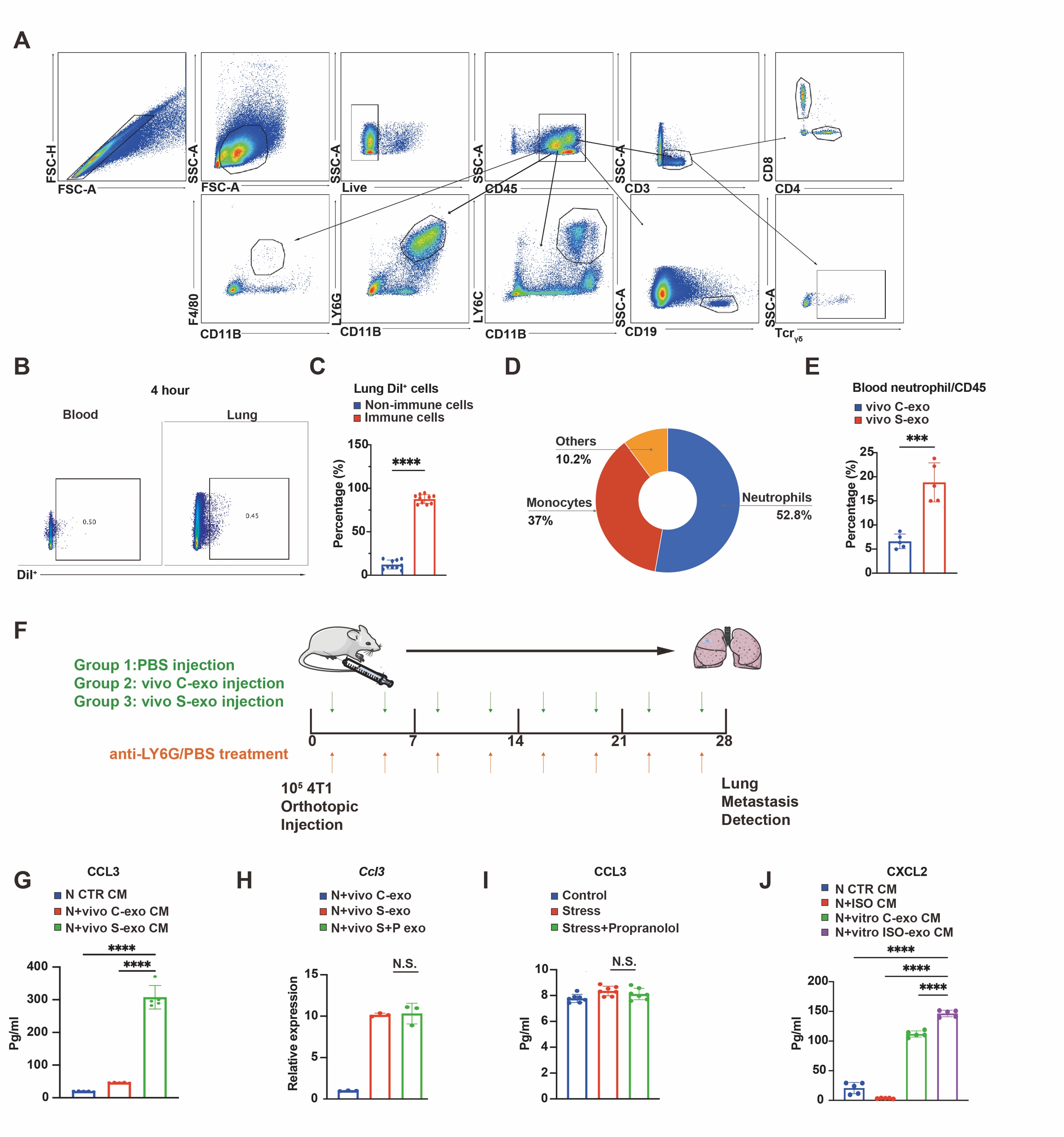


**Figure S4.**

**A**. Flow cytometry gating strategy used to define γδT cells (CD45^+^ CD3^+^ TCR_γδ_^+^), CD4^+^ T cells (CD45^+^ CD3^+^ CD4^+^ CD8^−^), CD8^+^ T cells (CD45^+^ CD3^+^ CD8^+^ CD4^-^ ), neutrophils (CD45^+^ CD11B^+^ LY6G^+^), monocytes (CD45^+^ CD11B^+^ LY6C^+^), macrophages (CD45^+^ CD11B^+^ F4/80^+^) and B cells (CD45^+^ CD19^+^). Dead cells were excluded with Zombie Red dye, lymphocytes and granulocytes were gated based on SSC-A versus FSC-A, and singlets were selected from the FSC-A versus FSC-H dot plot. **B**. Representative flow cytometry images of PB and lung exosome uptake 4 hours after injection of DiI^+^ exosomes. **C**. Flow cytometry quantification of pulmonary DiI^+^ exosome uptake by CD45^-^ cells and CD45^+^ immune cells (n=10). **D**. The representative proportion of different DiI^+^ immune cell types in the lungs after injection of DiI^+^ exosomes. **E**. Flow cytometry quantification of peripheral blood-infiltrating neutrophils 4 hours after injection of DiI^+^ vivo C-exo (n=5) or vivo S-exo (n=5). **F**. Schematic illustrating neutrophil depletion with anti-LY6G antibody and exosome injection in the mouse 4T1 breast cancer orthotopical model. **G**. ELISA analysis of CCL3 concentrations in the supernatant of naïve neutrophils treated alone, with vivo C-exo (n=5), vivo S-exo (n=5) respectively for 4h. **H**. qRT-PCR analysis of *Ccl3* expression of naïve neutrophils treated with vivo C-exo (n=3), vivo S-exo (n=3), vivo S+P-exo (n=3) for 4h. **I**. ELISA analysis of CCL3 concentrations in the lung supernatant of 4-week tumor-bearing mice in the control group (n=7), chronic stress group (n=7), and chronic stress & propranolol co-treated group (n=7). **J**. ELISA analysis of CXCL2 concentrations in the supernatant of naïve neutrophils treated alone, with ISO (n=5), vitro C-exo (n=5), vitro ISO-exo (n=5) respectively for 4 hours. The data are shown as mean ± SEM. ***: p < 0.001, ****: p < 0.0001.


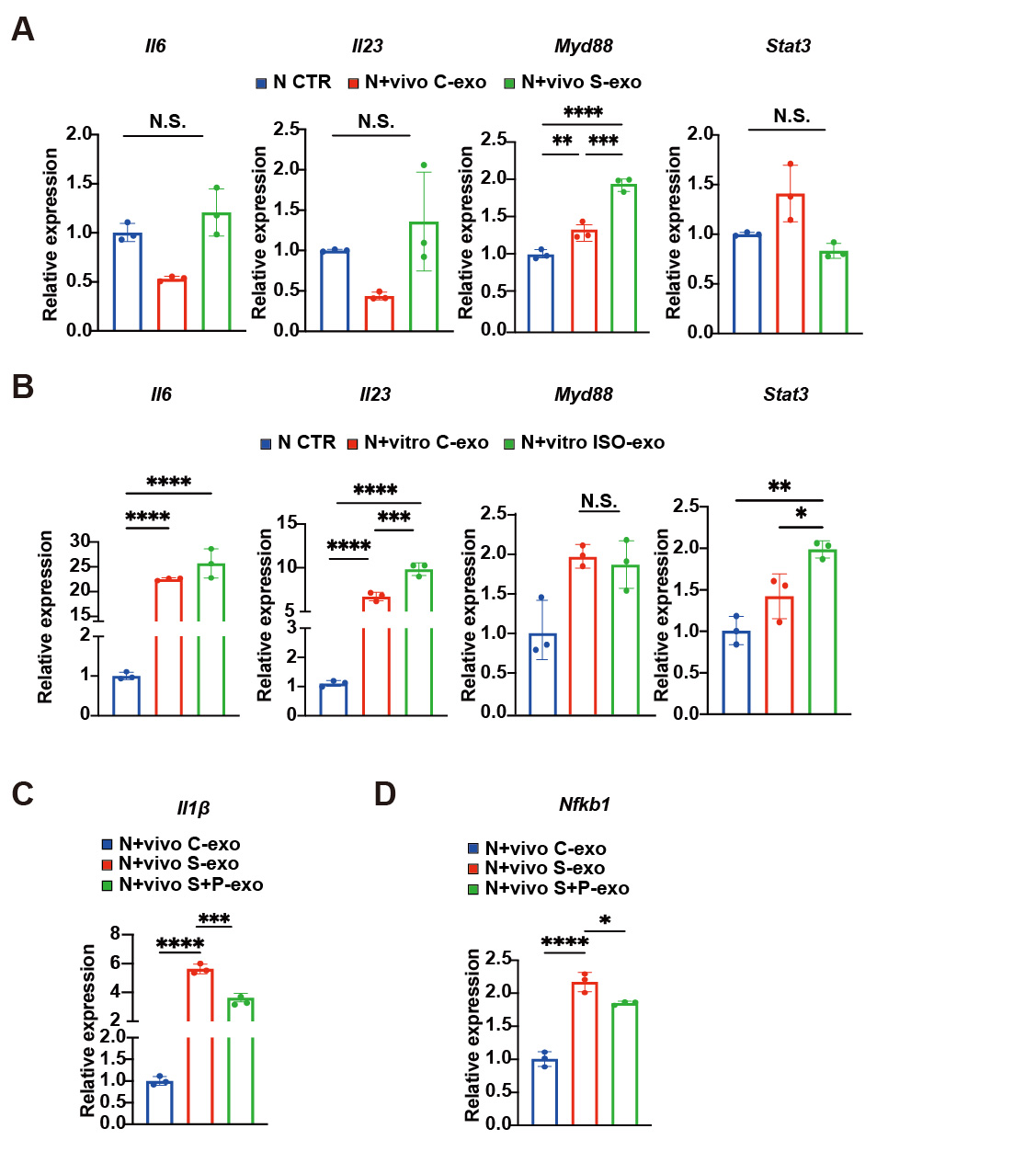


**Figure S5.**

qRT-PCR analysis was conducted to evaluate the expression levels of inflammation-related genes in naïve neutrophils treated alone (n=3), with vivo C-exo (n=3), vivo S-exo (n=3), vivo S+P-exo (n=3), vitro C-exo (n=3), vitro ISO-exo (n=3) respectively for 4 hours. The data are shown as mean ± SEM. *: p < 0.05, **: p < 0.01, ***: p < 0.001, ****: p < 0.0001.


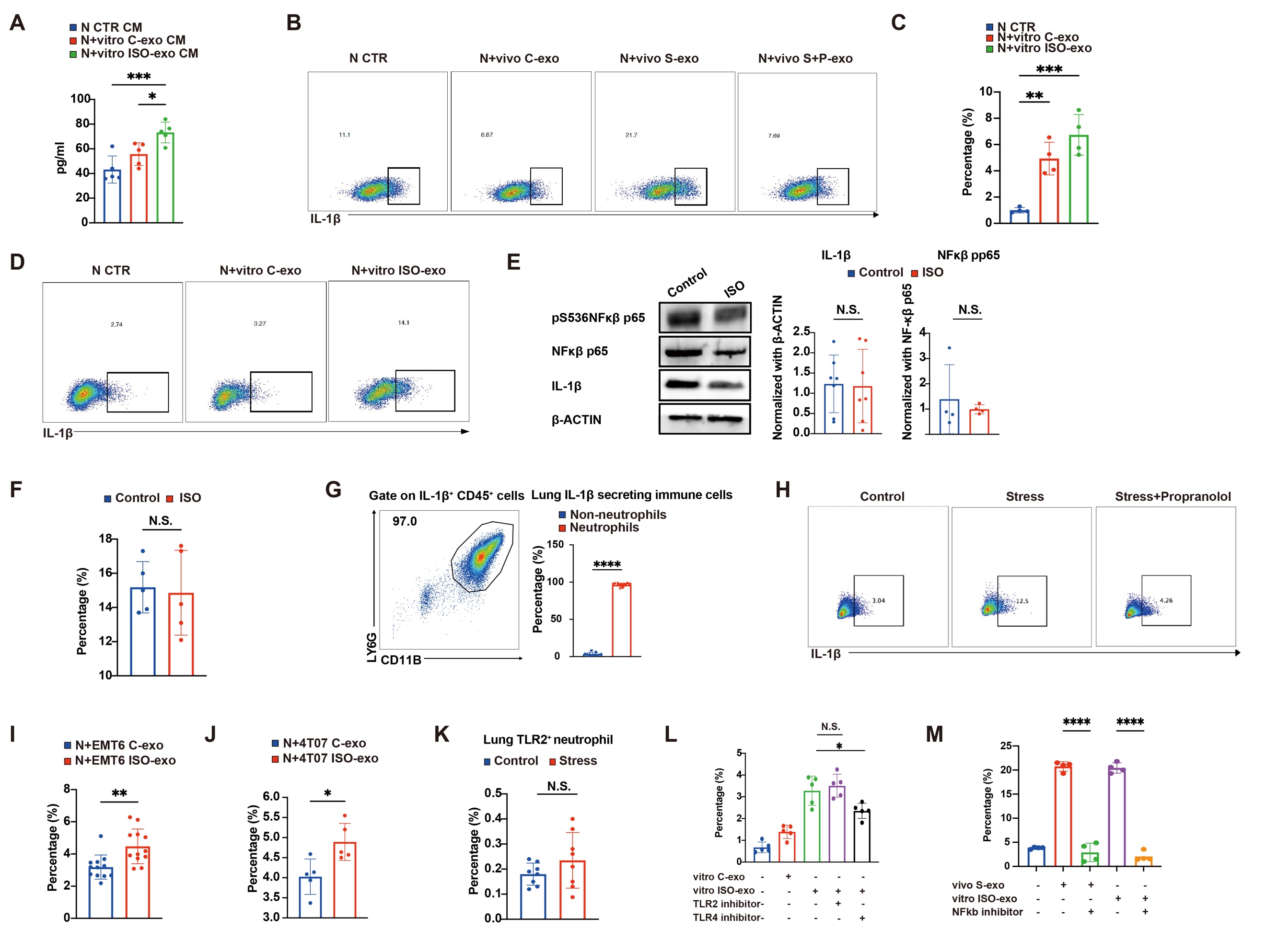


**Figure S6.**

**A**. ELISA analysis of IL-1β concentrations in the supernatant of naïve neutrophils treated alone (n=5), with vitro C-exo (n=5), vitro ISO-exo (n=5) respectively for 4 hours. **B**. Representative flow cytometry images illustrate the presence of IL-1β^+^ neutrophils in naïve neutrophils treated alone, with vivo C-exo, vivo S-exo, vivo S+P-exo respectively for 4 hours. **C**. IL-1β^+^ neutrophils quantification by flow cytometry in naïve neutrophils treated alone (n=4), with vitro C-exo (n=4), vitro ISO-exo (n=4) respectively for 4 hours. **D**. Representative flow cytometry images of IL-1β^+^ neutrophils in naïve neutrophils treated alone, with vitro C-exo, or with vitro ISO-exo for 4 hours. **E**. WB analysis was conducted to assess the expression of IL-1β (n=7) and NFκβ pp65 (n=4) in naïve neutrophils treated alone or with ISO for 4 hours. **F**. The quantification of IL-1β^+^ neutrophils by flow cytometry was performed in naïve neutrophils treated alone (n=5) or with ISO (n=5) for 4 hours. **G**. Flow cytometry quantification of IL-1β^+^ neutrophils as a percentage of total IL-1β^+^ CD45^+^ immune cells in the lungs of 4-week tumor-bearing mice in the chronic stress group (n=20). **H**. Representative flow cytometry images of lung-infiltrating IL-1β^+^ neutrophils of 4-week tumor-bearing mice from the control, chronic stress, and chronic stress & propranolol co-treated groups. **I**. Flow cytometry analysis of IL-1β^+^ neutrophils in naïve neutrophils treated with either EMT6 C-exo (n=12) or with EMT6 ISO-exo (n=12) for 4 hours. **J**. Flow cytometry analysis of IL-1β^+^ neutrophils in naïve neutrophils treated with either 4T07 C-exo (n=5) or with 4T07 ISO-exo (n=5) for 4 hours. **K**. Flow cytometry quantification of lung-infiltrating TLR2^+^ neutrophils in 4-week tumor-bearing mice in the control (n=8) and chronic stress groups (n=8). **L**. Flow cytometry quantification of IL-1β^+^ neutrophils in naïve neutrophils treated alone (n=5), with vitro C-exo (n=5), vitro ISO-exo (n=5), vitro ISO-exo and TLR2 inhibitor (n=5), vitro ISO-exo and TLR4 inhibitor (n=5) respectively for 4 hours. **M**. Flow cytometry quantification of IL-1β^+^ neutrophils in naïve neutrophils treated alone (n=4), with vivo S-exo (n=4), vivo S-exo and NFκβ inhibitor (n=4), vitro ISO-exo (n=4), vitro ISO-exo and NFκβ inhibitor (n=4) respectively for 4 hours. The data are shown as mean ± SEM. *: p < 0.05, **: p < 0.01, ***: p < 0.001, ****: p < 0.0001.


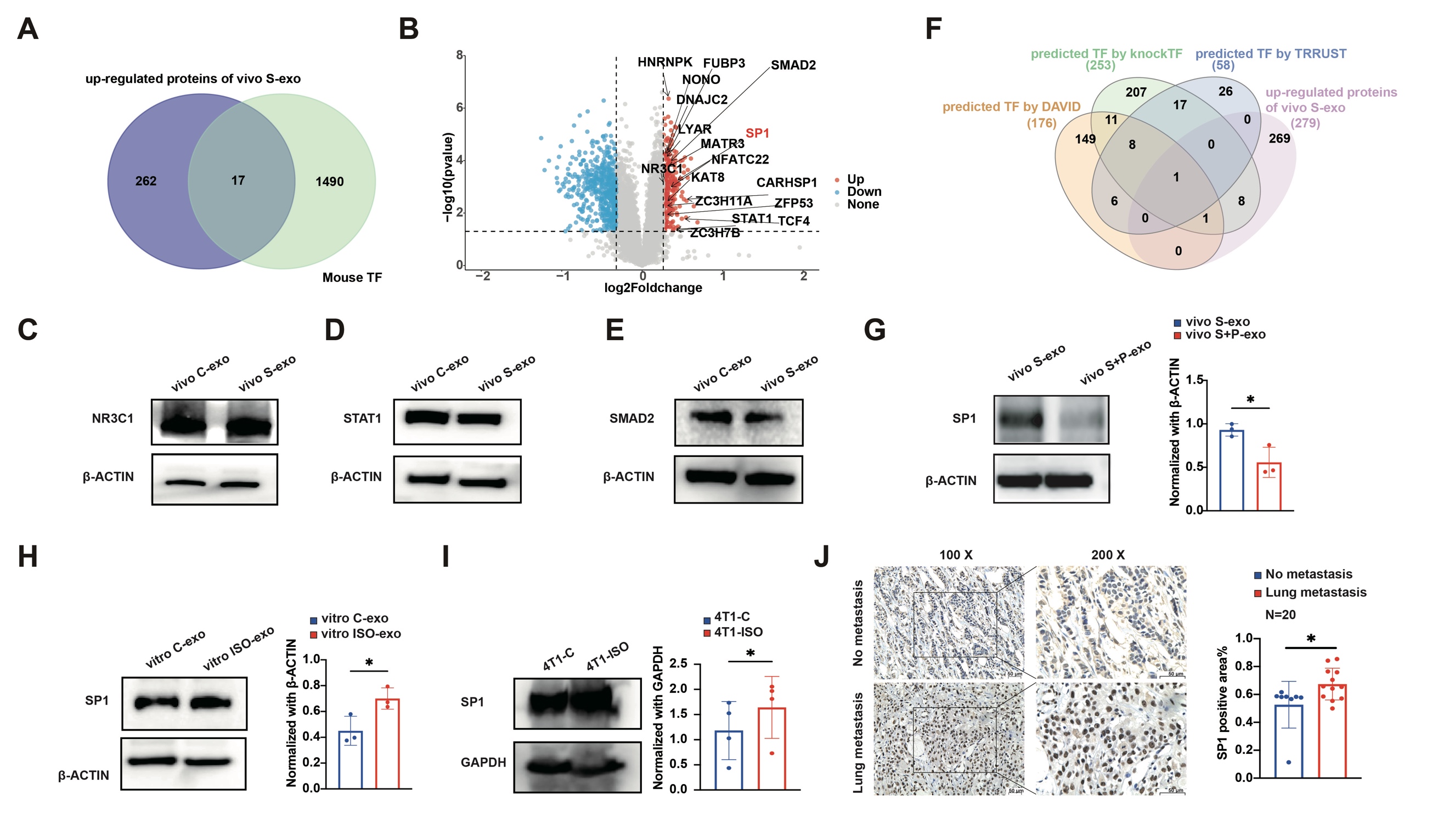


**Figure S7.**

**A**. Venn diagram depicting the up-regulated proteins of vivo S-exo identified by proteome analysis, compared to the list of mouse TFs. **B**. Volcano plot illustrating the proteome analysis results. Each dot in the volcano plot represents a protein, with those significantly up-regulated in vivo S-exo shown in red and those down-regulated in vivo S-exo shown in blue. Intersecting up-regulated TFs in vivo S-exo are indicated. Representative WB images showing the expression of NR3C1 (**C**), STAT1 (**D**), and SMAD2 (**E**) in vivo C-exo and vivo S-exo. **F**. Venn diagram showing the predicted TFs identified by DAVID, knock TF, and TRRUST databases, based on the up-regulated genes of pulmonary neutrophils in stressed mouse and the up-regulated proteins of vivo S-exo from proteome analysis. **G**. WB analysis of SP1 expression in vivo S-exo (n=3) and vivo S+P-exo (n=3). **H**. WB analysis of SP1 expression in vitro C-exo (n=3) and vitro ISO-exo (n=3). **I**. WB analysis of SP1 expression in 4T1 cells treated either without or with ISO (n=4). **J**. Quantification of SP1 immunohistochemical staining in breast cancer patients with different metastasis status (n=20 in total). The data are shown as mean ± SEM. *: p < 0.05, **: p < 0.01, ***: p < 0.001, ****: p < 0.0001.


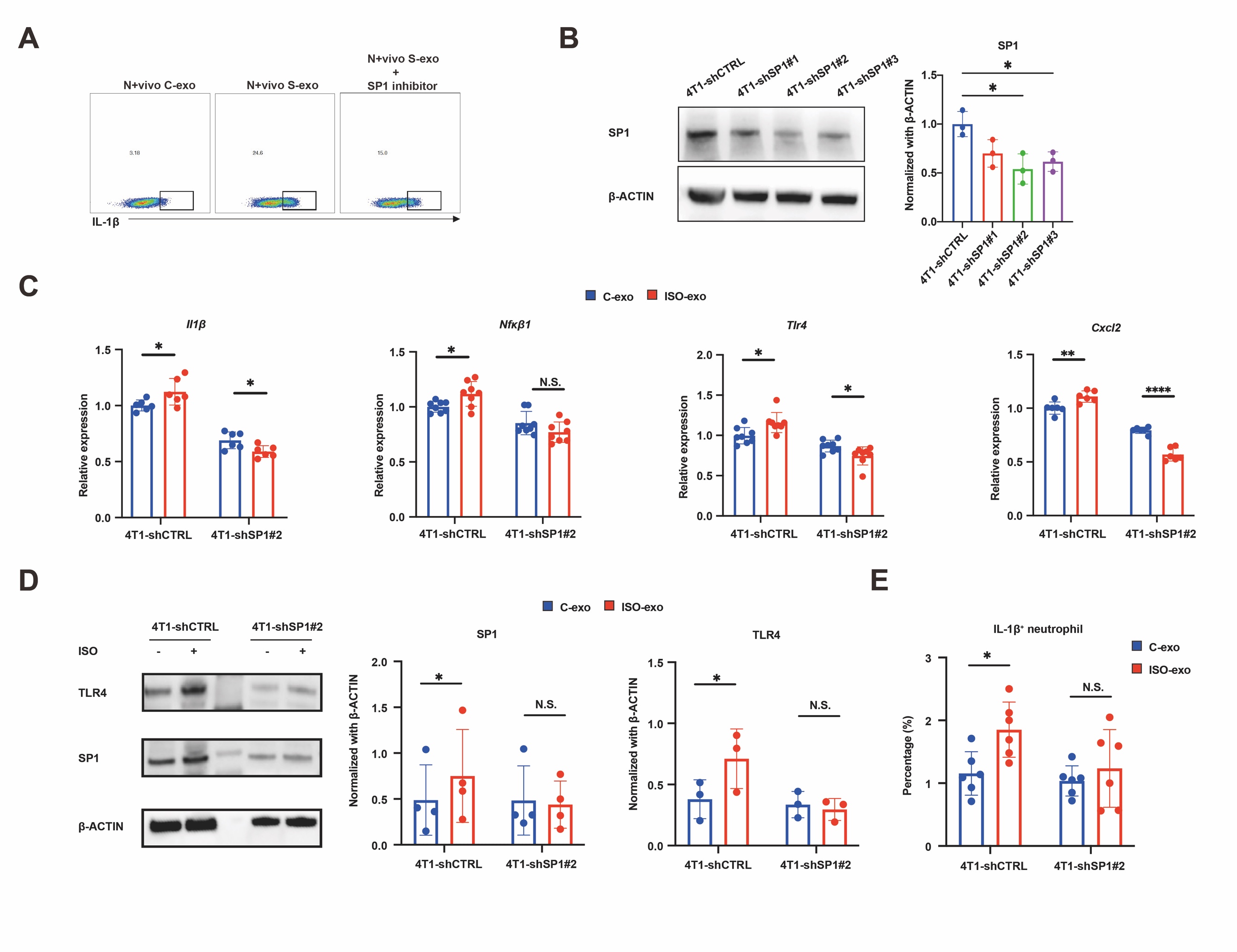


**Figure S8.**

**A**. Representative flow cytometry images of IL-1β^+^ neutrophils in BM-PMNs treated with vivo C-exo, vivo S-exo, or vivo S-exo combined with 500 nM mithramycin A respectively for 4 hours. **B**. WB analysis of SP1 expression in 4T1-shCTRL (n=3), 4T1-shSP1#1 (n=3), 4T1-shSP1#2 (n=3), and 4T1-shSP1#3 cells (n=3). BM-PMNs were treated with 4T1-shCTRL C-exo, 4T1-shCTRL ISO-exo, 4T1-shSP1#2 C-exo, 4T1-shSP1#2 ISO-exo for 4 hours, respectively. qRT-PCR analysis of the expression levels of *Il1β* (n=6), *Nfκβ1* (n=8), *Tlr4* (n=8), and *Cxcl2* (n=6) (**C**), WB analysis of the expression intensities of TLR4 (n=3) and SP1 (n=4) **(D),** and flow cytometry quantification of IL-1β^+^ neutrophils (n=6) **(E)** in the co-cultured neutrophils were performed. The data are shown as mean ± SEM. *: p < 0.05, **: p < 0.01, ***: p < 0.001, ****: p < 0.0001.


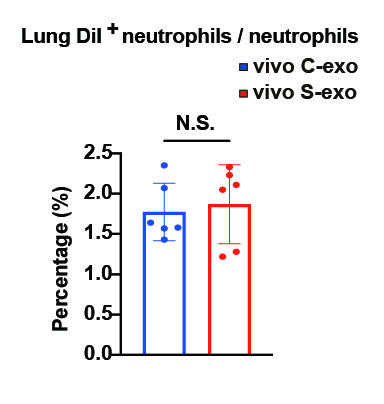


**Figure S9.**

The quantitative analysis of the proportion of DiI^+^ neutrophils in the lungs 4 hours post-injection of DiI-labeled vivo C-exo (n=6) or vivo S-exo (n=6).
